# Micro-RNA193a-3p Inhibits Breast Cancer Cell Driven Growth of Vascular Endothelial Cells by Altering Secretome and Inhibiting Mitogenesis: Transcriptomic and Functional Evidence

**DOI:** 10.3390/cells11192967

**Published:** 2022-09-23

**Authors:** Giovanna Azzarito, Lisa Kurmann, Brigitte Leeners, Raghvendra K. Dubey

**Affiliations:** 1 Department of Reproductive Endocrinology, University Hospital Zurich, 8952 Schlieren, Switzerland; 2 Department of Pharmacology & Chemical Biology, University of Pittsburgh, Pittsburgh, PA 15219, USA

**Keywords:** angiogenesis, vascular endothelial cells, Micro-RNA, MCF-7, secretome, conditioned medium, breast cancer, proliferation, migration, transcriptome, genes, DRGs

## Abstract

Breast cancer (BC) cell secretome in the tumor microenvironment (TME) facilitates neo-angiogenesis by promoting vascular endothelial cell (VEC) growth. Drugs that block BC cell growth or angiogenesis can restrict tumor growth and are of clinical relevance. Molecules that can target both BC cell and VEC growth as well as BC secretome may be more effective in treating BC. Since small non-coding microRNAs (miRs) regulate cell growth and miR193a-3p has onco-suppressor activity, we investigated whether miR193a-3p inhibits MCF-7-driven growth (proliferation, migration, capillary formation, signal transduction) of VECs. Using BC cells and VECs grown in monolayers or 3D spheroids and gene microarrays, we demonstrate that: pro-growth effects of MCF-7 and MDA-MB231 conditioned medium (CM) are lost in CM collected from MCF-7/MDA-MB231 cells pre-transfected with miR193a-3p (miR193a-CM). Moreover, miR193a-CM inhibited MAPK and Akt phosphorylation in VECs. In microarray gene expression studies, miR193a-CM upregulated 553 genes and downregulated 543 genes in VECs. Transcriptomic and pathway enrichment analysis of differentially regulated genes revealed downregulation of interferon-associated genes and pathways that induce angiogenesis and BC/tumor growth. An angiogenesis proteome array confirmed the downregulation of 20 pro-angiogenesis proteins by miR193a-CM in VECs. Additionally, in MCF-7 cells and VECs, estradiol (E2) downregulated miR193a-3p expression and induced growth. Ectopic expression of miR193a-3p abrogated the growth stimulatory effects of estradiol E2 and serum in MCF-7 cells and VECs, as well as in MCF-7 and MCF-7+VEC 3D spheroids. Immunostaining of MCF-7+VEC spheroid sections with ki67 showed miR193a-3p inhibits cell proliferation. Taken together, our findings provide first evidence that miR193a-3p abrogates MCF-7-driven growth of VECs by altering MCF-7 secretome and downregulating pro-growth interferon signals and proangiogenic proteins. Additionally, miR193a-3p inhibits serum and E2-induced growth of MCF-7, VECs, and MCF-7+VEC spheroids. In conclusion, miRNA193a-3p can potentially target/inhibit BC tumor angiogenesis via a dual mechanism: (1) altering proangiogenic BC secretome/TME and (2) inhibiting VEC growth. It may represent a therapeutic molecule to target breast tumor growth.

## 1. Introduction

Breast cancer (BC) is the most common malignancy, affecting one in eight women [1,2]. BC tumor progression is a dynamic process that involves interaction between cancer cells with multiple other cells within its microenvironment. Paracrine factors generated by BC cells and vascular endothelial cells (EC), lymphatic ECs, fibroblasts/stromal cells, or immune cells can interact to promote tumor growth and metastasis [3,4,5,6]. Hence, a better understanding of the interactions between BC cells with other cells in the tumor microenvironment (TME) is required to effectively target, tumor growth. Indeed, not all anti-cancer therapies are effective against BC and this resistance is in part, attributed to genetic mutations and epigenetic alterations [5,7]. There is growing consensus that targeting genetically stable cells and pro-growth factors within the TME may be more effective in treating BC/tumor progression. Indeed, molecules that target neo-angiogenesis, a key driver for tumor growth and cancer progression have been used clinically to block tumor growth [3,8,9,10]. Recently the focus has also expanded to target stromal/fibroblasts and immune cells as a means to restrict tumor progression and metastasis.

Crosstalk between cancer cells and vascular endothelial cells (VECs) plays an important role in BC and tumor neo-angiogenesis drives BC growth and metastasis [6]. Therapeutic molecules individually targeting BC cells or VECs have shown therapeutic success to some extent [3,7,10]. Hence, in-depth understanding of crosstalk between cells within the BC-TME may provide critical information to improve and target BC. We have recently shown that factors in BC cell secretome promotes VEC growth/angiogenesis [11]. Hence, identifying molecules that can target growth of both BC cells and VECs as well as alter BC secretome from pro- to anti-angiogenic characteristic may be effective in limiting tumor progression by inhibiting neo-angiogenesis.

MicroRNAs (miRNA) are small (~22 nucleotides) endogenous non-coding RNAs that regulate gene expression by binding to the 3′-untranslated regions (UTR) of the target mRNA of protein-coding genes [12]. These actions modulate a wide range of biological processes, including proliferation, differentiation, apoptosis, invasion and migration, which play an important role in the pathophysiology of several diseases [13]. Importantly, studies have shown the active involvement of microRNAs (miRNA) in biological processes associated with BC [14]. Evidence suggests that miRNAs are often deregulated in various cancers [15,16], including BC [17], and play important roles in tumorigenesis by modulating the expression of their targets. They are involved in regulating tumor invasion, metastasis and progression by modulating suppressor genes and/or oncogenes [18]. The ability of miRNA to silence multiple targets has made their regulation an important target for the development of new pharmacological strategies in cancer treatment [19], with many miRNAs currently in phase-II clinical trials [20,21].

In the present study, we focused on the involvement of human miRNA193a-3p in MCF-7 secretome and estradiol driven growth of VECs. MiR-193a is located on human chromosome 17q11.2 and is a member of the miR-193 family including miR-193a, miR-193b, and miR-193c that can prevent proliferation and cell cycle modulation [22]. During miRNA biogenesis, two mature miRNAs (arms) are generated from pre-miR-193a: miR-193a-3p and miR-193a-5p. Both miRNAs are downregulated in various cancers, and they exhibited tumor suppressive properties mediated via different mechanisms [23,24,25]. Overexpression of miRNA193a-3p inhibits cancer cell proliferation and induces apoptosis, which makes it an attractive molecule for cancer treatment, as well as a biomarker in cancer. In order to identify better therapies for BC, it is essential to understand its pathogenesis in greater depth. In the present study, we evaluated the effects of miRNA 193a-3p on the pro-growth crosstalk between BC cells and VECs (key cells within BC TME), and the underlying mechanisms.

Pro-growth role of estrogen in ER positive BC is well established [23,24]. Moreover, estrogen directly regulates genes and microRNAs via estrogen receptors or by activating intracellular signaling cascades, leading to altered gene expression [12]. Indispensable role for miRNAs in mediating estrogen responses through various cellular processes has been documented in estrogen sensitive cells, for example: osteoblasts [25], mesenchymal stem cells [26] and osteocytes [27]. Micro RNAs are also reported to be involved in estrogen regulatory networks [28]. In MCF-7 cells, E2 upregulated the expression of 21 miRNAs and downregulate the expression of 7 miRNAs [29]. Based on the important role played by both miRNAs and E2 in tumor growth, the identification of specific miRNAs regulated by E2 may provide valuable insights to improve breast cancer treatment. In fact, the expression of miRNA inhibited by E2 modulates cancer cell proliferation and apoptosis and consequently blocks tumor progression [30]. Importantly, miRNA193a-3p levels are downregulated in BC [19,31]; epigenetic alterations in miR193a-3p promotes HER2 positive BC aggressiveness [32]; and miR193a inhibits BC proliferation and metastasis [31,33]. However, the underlying mechanism(s) remain unclear. The fact that estrogen is associated with BC and induces mitogenic actions in BC cells [33], whereas, miR193a-3p inhibits BC cell growth [31]. We hypothesize that estrogen may, in part, mediate its growth promoting actions in ER positive BC cells i.e., MCF-7 by downregulating miR193a-3p. Since estrogen also induces growth of VECs [34], it is feasible that estrogen induces VEC growth by downregulating miR193a-3p. Based on our recent finding that MCF-7 secretome induces VEC growth [11], we hypothesize that ectopic expression of miR193a-3p in MCF-7 cells may abrogate the release of pro-growth factors in secretome and block MCF-7 driven growth of VECs.

The overall aim of the present study was to delineate the direct and indirect mechanisms by which miR193a-3p may induce its anti-angiogenic activity in BC, specifically how miR193a-3p alters BC cells (MCF-7) induced growth of VECs. Using MCF-7 secretome/conditioned medium from BCs pre-transfected with miR193a transfected BCs we assessed the impact of MCF-CM on VEC growth, signal transduction pathways (ERK1/2 and Akt phosphorylation), gene expression (microarrays/transcriptomic analysis) and angiogenic protein arrays. Since MCF-7 cells are estrogen responsive BC cells, we also assessed the direct modulatory effects of miR193a-3p on estradiol-induced growth of both VECs and MCF-7 cells. Moreover, we assessed the effects of estradiol on miR193a-3p expression in VECs and MCF-7 cells, and tested the effects of miR193a-3p on MDA-MB231(ER independent BC cells) secretome induced VEC growth. Finally, we developed and utilized a 3D spheroid model of MCF-7 plus VECs to investigate the impact of miR-193a-3p on their crosstalk-induced growth.

## 2. Materials and Methods

### 2.1. Cell Culture

Human Umbilical Vein Endothelial Cells (VECs) were purchased from Lonza (Walkersville, MD, USA (CC-2517)). Human VECs were cultured in Collagen (rat tail, 5 μg/cm^2^) coated flasks under standard tissue culture conditions (37 °C, 5% CO_2_) in growing media: EBM-2 (Endothelial Basal Medium-2) supplemented with Glutamax (1x), antibiotic-antimycotic (AA: 100 μg/mL streptomycin, 100 μg/mL penicillin and 0.025 μg/mL amphotericin B), LSGS (2% *v*/*v* FCS, 1 µg/mL hydrocortisone, 10 ng/mL human Epidermal Growth Factor, 3ng/mL human basic Fibroblast Growth Factor, 10 µg/mL heparin) and 10% FCS (Fetal Calf Serum). MCF-7 human breast cancer cell line (mammary epithelial cells) was provided from Dr André Fedier (Clinic for Gynecology, University Hospital Zurich). MCF-7 cells were cultured in DMEM/F12 medium supplemented with Glutamax (1x), antibiotic-antimycotic (AA 100 μg/mL streptomycin, 100 μg/mL penicillin and 0.025 μg/mL amphotericin B) and 10% FCS.

### 2.2. Conditioned Media (CM) Formation

**Figure 1:**
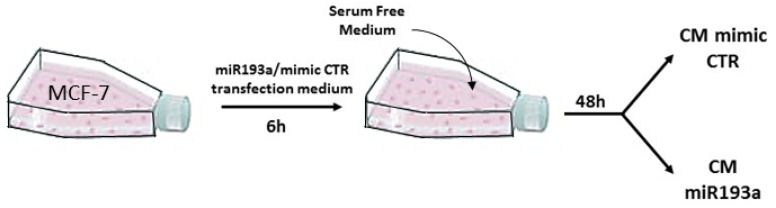
A representative scheme of Conditioned media formation. MCF-7 cells are grown on 75 cm^2^ tissue culture flasks and transfected with miR193a-3p and its relative mimic control. Medium was replaced with serum free medium and collected after 48 h. Same approach was used to collect CM from MDA-MB-231 BC cells.

When MCF-7 or MDA-MB-231 cells reach 70% confluence in 75 cm^2^ tissue culture flasks, they were transfected with miRNA/mimic CTR Lipofectamine 2000. After 6 h the transfection medium was replaced with serum free medium (EBM-2, Glutamax, antibiotic-antimycotic solution) for 48 h (Figure 1). The supernatant containing all factors secreted by the cells, was harvested from the cultures, centrifuged (5 min, 1000× *g*, at RT) and filtered through 0.2 µm syringe filters. The resulting conditioned media (CM) was aliquoted and stored at −80 °C or directly used.

### 2.3. Spheroid Formation

Spheroids were prepared as we previously described [35] using spheroid microplate method or hanging droplet method. Briefly, 5 × 10^2^ cells/well (for proliferation studies) or 5 × 10^3^ cells/well (Immunohistochemistry) in growth or treatment medium were seeded in 96-U bottom low-attachment plates (Thermo Scientific, Roskilde, Denmark, Nunclon sphera, 174925). Hanging drop was carried out in the petri dish lid by dropping 15 µL of cell suspension (3.4 × 10^5^ cells/mL). Subsequently the lid was inverted, and the cells forced to accumulate at the bottom of the drop and and aggregate to form spheroids. Plate/dish was placed in a humidified atmosphere of 5% CO_2_ at 37 °C and incubated for 96 h. To form multicellular spheroids, MCF-7 and VECs were mixed in a ratio 1:1.

### 2.4. Transfection with miRNA

Cells were plated in the respective growth medium and allowed to attach and recover for 48 h. Lipofectamine 2000 (0.17%), miR193a (human miRNA (10 nmole), final concentration of 25 nM) and the relative mimic control (miRNA Negative Control, mimic #1 (10 nmole)) were diluted in serum and antibiotics free medium (DMEM-F12 (for MCF-7) or EBM-2 (For VECs)) and incubated for 5 min at RT. The solutions were equally mixed and incubated for 20 min at RT to allow the miRNA oligonucleodites:lipofectamine complex to form. Cells are washed with HBSS (containing Ca^2+^ and Mg^2+^), rinsed with serum and antibiotics free medium and the oligonucleodites:lipofectamine complex was also added. After 6 h in standard tissue culture conditions (37 °C, 5% CO_2_) the transfection medium was replaced with normal growing or treatment medium. To obtain transfected spheroids, cells were transfected separately in monoculture, trypsinized, counted and seeded in a 1:1 ration. The miR-193a-3p used is a hsa-miR-193a-3p with the following sequence 5′–AACUGGCCUACAAAGUCCCAGU–3′ (FASTA sequence shown in Supplementary File Figure S1).

### 2.5. Cell Proliferation Assay

Cell proliferation assay was performed by counting cell number. Cells were seeded in 24-well plate and after 48 h, the growth was arrested in starving medium (EBM-2, Glutamax, antibiotic-antimycotic solution, and 0.4% steroid free FCS) for 6 h or overnight. It was then replaced with CM (supplemented with 0.4% FCS) or treatment/transfection medium (2.5% steroid free FCS medium). The cells were trypsinized and counted using Coulter Counter (Coulter Electronics, Luton, UK).

Spheroid proliferation assay was performed by calculating total spheroid area. Spheroids are grown in 96-well U-bottom plates with cells pretreated with 10 nM E2 or cells pre-transfected with miRNA. After 96 h in culture, bright field pictures of spheroids were taken with an inverted microscope. Images of the spheroids were subsequently processed to 300 pixels/inch and analyzed using ImageJ software, as previously described [35].

### 2.6. Migration Studies

VECs were plated in a collagen-coated 24-well plate (Biocoat Collagen I Cellware, Corning, Kennebunk, ME, USA, 354408) and grown to confluence. Using a yellow pipette tip, a scratch was made; subsequently, the cells were washed once with HBSS (with Mg^2+^ and Ca^2+^) and CM (in presence of 0.4% FCS) was added. Images of the scratch were photographed using Olympus inverted microscope at time 0 h and after 24 h. Would closure area was determined using ImageJ software (ImageJ 1.52a, Wayne Rasband, National Institutes of Health, Kensington, MD, USA) and calculated accordingly: (area T0–area T24)/area T0.

### 2.7. Microvessel Formation Assay

An angiogenesis μ-slide was coated with ice-cold Matrigel solution and incubated at 37 °C for at least 30 min to allow the Matrigel to solidify. The trypsinized VECs were counted and suspended in 1 mL per each CM at a density of 80,000 cells/mL. They were supplemented with 0.4% FCS; the cells were incubated 30 min prior to the transfer of 50 µL (4000 cells) on top of the gel. The μ-slide was incubated at 37 °C for 5h to allow cells to form micro-vessels. Images of the tubular structures were taken for each well at using an Olympus inverted microscope. The quantification was done measuring micro-vessels’ length using the Olympus Xcellence Pro software (Volketswil, CH, Switzerland).

### 2.8. Western Blot

Endothelial cells were seeded in 35-mm tissue culture dishes and allowed to adhere. The attached cells were starved by feeding EBM-2 medium containing 0.4% steroid-free serum for 7 h. Subsequently the cells were cultured in either CM miR193a or CM mimic CTR. After 45 min incubation under cell culture conditions the cells, were washed with cold PBS and lysed. The samples were homogenized and stored at −20 °C until further use. Protein concentration was measured using the Pierce BCA Assay Kit (Thermo Fisher). The cell extracts were separated by 10% SDS-polyacrylamide gels electrophoresis and transferred to a nitrocellulose membrane. The membrane was blocked in 5% milk and incubated overnight with the primary antibody. After three washing steps with 1% milk, the membrane was incubated with the secondary antibody for 1 h and the bands detected with the CAWOMAT 2000 IR film developer, pre-exposed to Super Signal West Dura Luminol Substrate.

### 2.9. Microarray

For microarray analysis, cells were seeded in 35-mm dishes in normal growth medium and allowed to attach. Subsequently, VECs were starved and cultured in CM (with 0.4% FCS) for 20 h. RNA isolation was performed using the Quick-RNA MiniPrep Kit (ZymoResearch, Irvine, CA, USA, R1055) and quantified using Tecan Spectrofluorometer reader (Infinite 200 NanoQuant). The samples were frozen at −80 °C and microarray analysis was performed using Affymetrix Clariom S Assay, human (Applied Biosystems by Thermo Fisher Scientific Inc., Waltham, MA, USA, 902927). For transcriptome analysis, fragmented biotin-labeled ds cDNA was hybridized to Clariom™ S arrays (Applied Biosystems by Thermo Fisher Scientific Inc., Waltham, MA, USA). For scanning, the Affymetrix Gene-Chip Scanner-3000-7G was used, and the image and quality control assessments were performed using GeneChip Command Console Software (GCC) v5.0 (Life Technologies, Santa Clara, CA, USA). Transcriptome analysis was conducted at the transcriptomic core facility at the Center for Molecular Medicine Cologne (CMMC). Differentially regulated genes were determined with the Transcriptome Analysis Console (TAC, Applied Biosystems by Thermo Fisher Scientific Inc., Waltham, MA, USA) based on a fold change cut-off of ± 1.5 and FDR *p*-value < 0.05. Pathway analysis was performed using Enrichr website. The microarray data were deposited in the public Gene Expression Omnibus (GEO) database under the accession no. GSE189084 (Available online: https://www.ncbi.nlm.nih.gov/geo/query/acc.cgi?acc=GSE189084) (accessed on 14 July 2022).

### 2.10. Quantitative RT-PCR

For microarray results validation, quantitative real-time polymerase chain reaction (qRT-PCR) was performed. VECs were cultured in CM and RNA isolation was performed as described in the Microarray section above. RNA concentration was determined by measuring the absorbance at 260 nm. 0.5 ug of total RNA was used for reverse transcription of each sample using the RT^2^ First Strand Kit (Qiagen) according to manufacturer’s instruction. Gene expression and detection was performed using a Bio-Rad CFX96 Real-Time PCR Detection System using Custom RT^2^ Profiler PCR Arrays in a 96-well-plate format according to the manufacturer’s protocol. The PCR reaction was run at 95 °C for 10 min followed by 40 cycles of 95 °C for 15 s and 60 °C for 1 min. As internal control, we used GAPDH and LDHA. The experiment was performed once in triplicates and the relative gene expression was calculated using the 2−ΔΔCt method.

### 2.11. Angiogenesis Proteome Array

To assess changes in angiogenesis-related proteins in VECs exposed to CM from miRNA193a-3p transfected MCF-7 cells (CM miRNA), we employed Proteome Profiler Human Angiogenesis Array Kit (R&D Systems, Minneapolis, MN, USA, ARY007). Cells were seeded in 60 mm tissue cultures dishes (~7 × 10^5^ cells/dish), starved for 6 h and cultured in CM miRNA (0.4% FCS) for 48 h. Following treatment, the cells were dislodged with trypsin and centrifuged. The pellet was lysed in lysis buffer 17 (R&D Systems, Minneapolis, MN, USA) supplemented with glycerol (10%) aprotinin, leupeptin and pepstatin (10 µg/mL each). The lysates were mixed by gentle rocking at 4 °C for 30 min and subsequently centrifuged for 5 min. The supernatant was transferred into clean tubes and frozen at −80 °C until further processing. The ready-to-use membranes were incubated o/n with equal amounts of samples (200 µg in 1.5 mL). The detection of following proteins was performed according to the manufacturer’s instructions. For the exposure of the membranes Amersham Hyperfilm ECL (GE Healthcare Limited, Buckinghamshire, UK) were used in a CAWOMAT 2000 IR film developer (Wiroma AG, Niederscherli, CH, Switzerland). Average signal of the pair of duplicate spots was determined using ImageJ software after background subtraction.

### 2.12. Immunohistochemistry

Spheroids were collected after 96 h of culture and fixed in 4% paraformaldehyde (PFA) for 1 h at RT. Supernatant was removed and after a washing step with PBS, spheroids were embedded in 2% Nobel Agar (BD Difco) solution (in PBS) and a short string in the middle of the agarose solution was added for an easy removal of the plug from the tube. The agarose was solidified, and the samples were ready for subsequent dehydration, paraffin embedding, sectioning, transfer onto the microscope slides, and IHC staining. Histological staining was performed with hematoxylin and eosin (H&E) for general evaluation of the cell cultures, with Ki67 (dilution 1:300) as proliferative marker and CD31 (dilution 1:200) as a specific marker for endothelial cells. Bright field images were taken using Leica microscope (M205 FA) and the quantification was made using ImageJ software. The Color Deconvolution ImageJ plugin implements was used to unmix dyes in the images.

### 2.13. Statistical Analysis

Data are expressed as mean ± SD of three independent experiments. Statistical calculations were run in R-studio. After the normal distribution was proved via Shapiro–Wilk test, a parametric test was performed with ANOVA analysis followed by Tukey’s HSD multiple pairwise comparisons. If the normality test was not passed, a non-parametric test was performed with Kruskal–Wallis rank sum test and subsequent pairwise Wilcoxon-test with Benjamini–Hochberg corrections.

## 3. Results

### 3.1. miR193a-3p Inhibits Serum-Induced Growth of MCF-7 Cells and VECs

**Figure 2:**
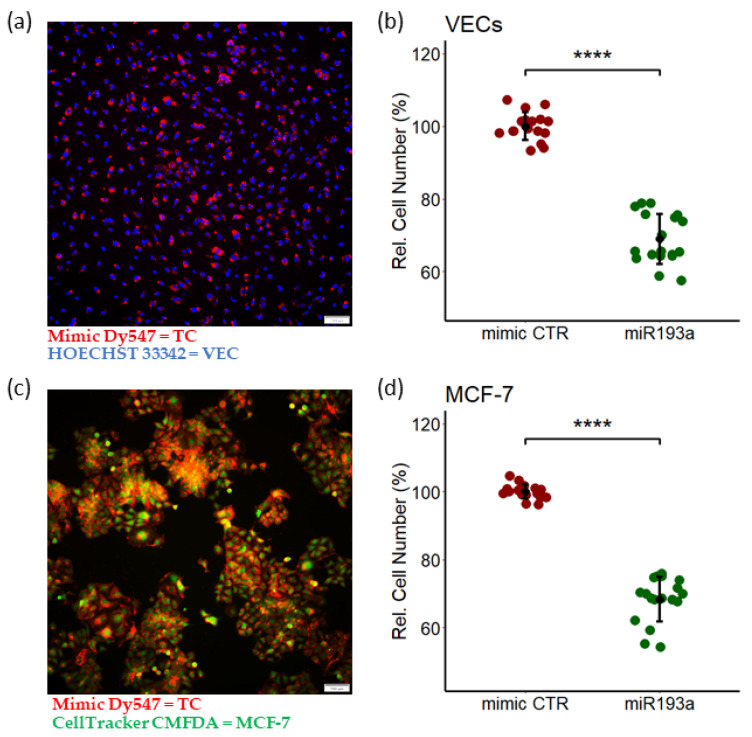
miR193a overexpression inhibits VEC and MCF-7 cell growth. Representative photomicrographs showing transfection efficiency of miR193a in VECs (**a**) and MCF-7 cells (**c**). Photomicrograph depicts fluorescence microscopy image of VECs transfected with Mimic Dy547. Red: Mimic Dy547; blue: HOECHST 33342; green: CellTracker Green CMFDA; TC: Transfected cells. Scale bar, 100 μm. VECs (**b**) and MCF-7 cells (**d**) were transfected with control mimic or miR193a and after 3 days cell proliferation was assessed by cell counting. Experiments were performed at least 3 times in triplicates. *p* < 0.00001 **** compared to the respective control.

Several miRNAs associated with cancer have been identified [36]. In particular, miRNA 193a-3p (miR193a) seems to play important role in BC [24,34,37]. To test whether miR193a-3p prevents growth induced by other mitogens, we assessed its impact on steroid free serum induced growth of VECs and MCF-7 cells. Both cell types were transfected with miR193a-3p and allowed to grow for 3 days in medium containing 2.5% steroid free fetal calf serum. As shown in Figure 2a,c, we observed high transfection efficiency (>90%) with miR-193a-3p in both VECs and MCF-7 respectively. Moreover, using this transfection protocol, a significant inhibition of VEC and MCF-7 cell growth (assessed by cell counting), was observed in cells transfected with miR193a-3p (Figure 2). Compared to miR mimic control, miR193a-3p inhibited growth of MCF-7 cells and VECs by 31.7% ± 2 and 31% ± 6, respectively (Figure 2b,d). These results demonstrated that miR193a has inhibitory effects of VECs and MCF-7 cells growth.

### 3.2. Effect of miR193a-3p Conditioned Media on VECs

**Figure 3:**
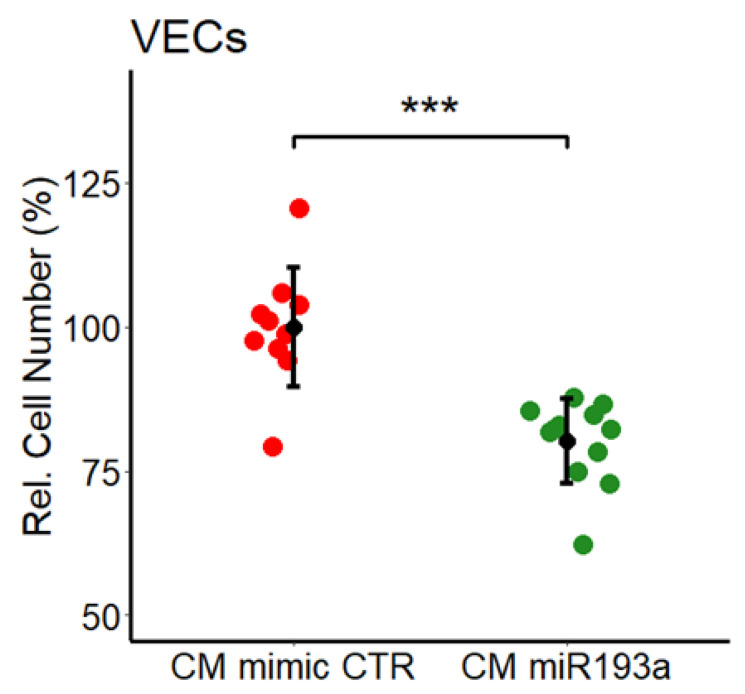
Secretome/CM from miR193a-3p transfected MCF-7 cells inhibits VEC proliferation. VECs were cultured in CM for 48 h and cell proliferation was assessed by cell counting. Figure shows the effects of CM miR193a produced by MCF-7 cells pre-transfected with miRNA193a or mimic CTR. Cell proliferation was inhibited, from a stimulatory effect of 176% in CM CTR to 20% inhibition in VECs treated with CM miR193a. Experiments were performed at least 3 times in triplicates or quadruplicates and the results are presented as mean ± SD. *p* < 0.001 *** compared to the respective control.

Growing evidence suggests that miRNA193a acts as a tumor suppressor in some malignancies [37]. Since the tumor microenvironment contributes to cancer/tumor progression, together with our recent finding that MCF-7 secretome induces VEC growth [11], we hypothesize that miR193a-3p may block MCF-7 driven growth of VECs by altering the secretome. To test our hypothesis, we collected secretome/conditioned medium (CM) from MCF-7 cells that were pre-transfected with miR193a-3p (CM miR193a) or with the relative mimic control (CM mimic CTR) (Figure 3). CM model has been widely used to assess cell–cell crosstalk and the impact of paracrine factors in modulating tumor growth. Here we used CM from MCF-7 cells that were pre-transfected with miR193a-3p or miR control to assess whether miR193a-3p alters MCF-7 secretome action on VEC growth. Cell proliferation, was assessed by counting VECs exposed to mimic control CM or miR193a-3p CM for 48 h of culture. In contrast to our previous finding [11], that CM from MCF-7 cells promotes robust growth (an increase of 76% ± 23.2) in VECs exposed for 48 h to MCF-7 secretome (CM CTR; Figure 3). However, the pro-growth effects of MCF-7 secretome were reversed in CM collected from MCF-7 cells pre-transfected with miR193a-3p failed to induce growth and inhibited VEC growth by 20% ± 7.3. Our results demonstrate that CM miR193a alters the growth promoting paracrine factors in MCF-7 secretome from pro-growth to anti-growth (Figure 3). The possibility that leakage of miR193a-3p from cells into the CM contributes to these effects can be ruled out, as no RNA was detectable in the CM of miR193a-3p-transfected cells. These findings suggest that the soluble factors in CM secreted by pre-transfected MCF-7 cells inhibit VEC proliferation.

### 3.3. Effect of miR193a-3p Conditioned Media on cell Migration/Wound Closure

**Figure 4:**
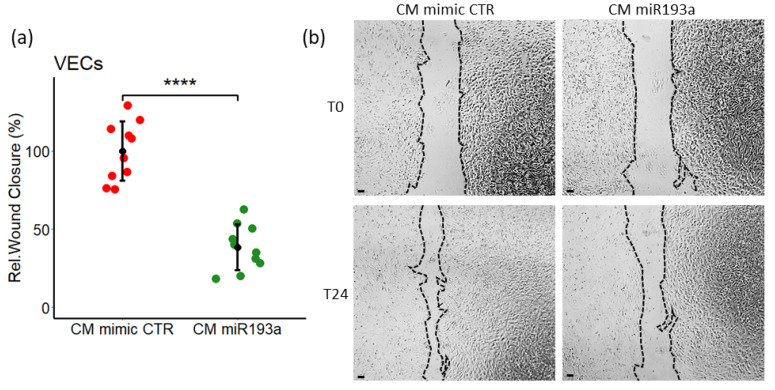
Secretome/CM from miR193a-3p transfected MCF-7 cells inhibits VEC migration/wound closure. Cell migration, was investigated using wound closure assay in confluent VECs monolayer. (**a**) CM from MCF-7 cells pre-transfected with miR193a inhibits wound closure as compared to CM from mimic CTR. All treatments were done in presence of 0.4% FCS and after the scratch was made. (**b**) Representative images of scratch wounds at time 0 (T0) and after 24 h (T24). Scale bar, 200 μm. Experiments were performed at least 3 times in triplicates or quadrupli-cates presented as mean ± SD. *p* < 0.0001 ****, compared to the respective control.

Cell migration may be a mechanism responsible for metastasis. Hence, we further investigated the impact of miRNA-CM activity on VEC migration using the wound-healing assay. As shown in Figure 4, In contrast to our previous findings [11] where CM from MCF-7 cells induced VEC migration by 50%, CM from MCF-7 cells pre-transfected with miR193-3p (CM miR193a) inhibited cell migration by 62% ± 14.5, as compared to their relative controls (Figure 4). No RNA/miR was detectable in CM from MCF-7 cells pre-transfected with miR193a, suggesting that the reversal of wound closure effects of CM CTR by CM miR193a were due to miR193a-3p alteration in the production of soluble factors in the MCF-7 secretome.

### 3.4. Effect of miR193a-3p Conditioned Media on Tube/Capillary Formation by VECs

**Figure 5:**
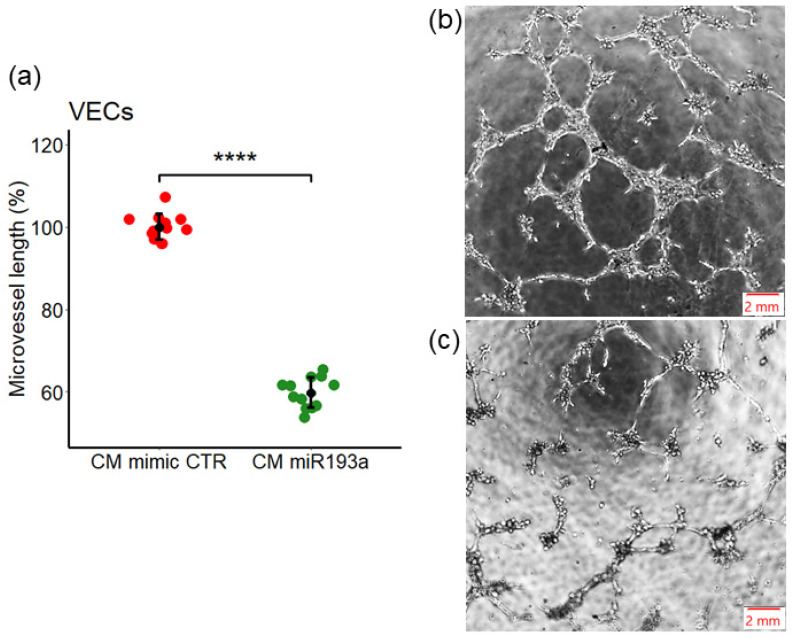
MCF-7 CM-induced VEC capillary formation are abrogated in CM from miR193a-3p transfected MCF-7 cells. Tube formation was investigated using Matrigel-based assay. Cells were incubated for 30 min with CM miRNA in 0.4% FCS before plating on Matrigel. Cells were allowed to form tube-like structures for 5 h. (**a**) Tube length was measured microscopically and compared with the respective mimic control. Experiments were performed at least three times in triplicates, and the values are expressed as mean ± SD, **** *p* < 0.0001. Photomicrographs depict representative images for each condition: CM mimic CTR (**b**) and CM miRNA193a (**c**). Scale bar, 2 mm.

Angiogenesis facilitates invasion, growth, and progression in a variety of tumors. Hence, we assessed the impact of MCF-7 CM miRNA on angiogenesis by studying capillary formation. As shown in Figure 5a, MCF-7 CM miR193a inhibited micro-vessel formation by 40% ± 3.7 as compared to its relative CTR. Representative photomicrographs in Figure 5b,c depict capillary formation and branching in VECs exposed to mimic control (CM mimic CTR), and miRNA193a (CM miR193a) CM from MCF-7 cells respectively. These results demonstrate that secretome from miR193a-3p transfected MCF-7 cells, significantly inhibits capillary formation by VECs.

### 3.5. Secretome/CM from miRNA193a-3p Transfected MCF-7 Inhibits VEC Growth by Downregulating ERK1/2 and PI3K-Akt Pathways

**Figure 6:**
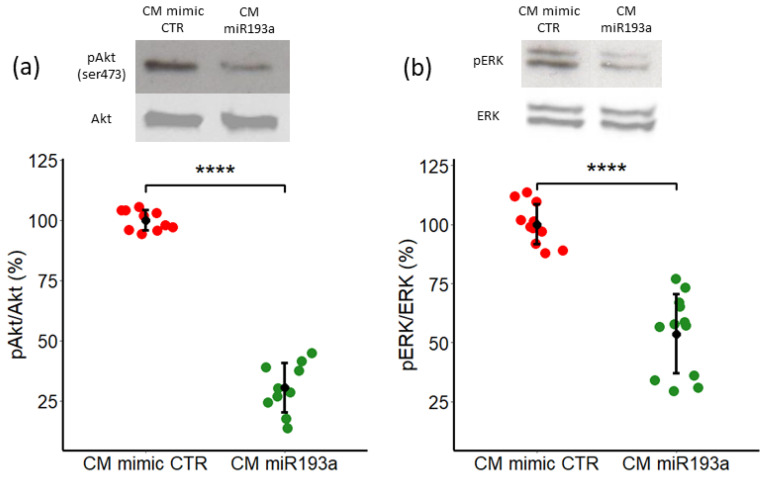
Secretome/CM from miR193a-3p transfected MCF-7 cells inhibits PI3k/Akt and ERK1/2 phosphorylation in VECs. Representative Western Blots and graphs depicting the effects of secretome from miR193a-3p transfected MCF-7 cells on Akt (**a**) and ERK1/2 (**b**) phosphorylation in VECs, after culturing in CM miR193 for 45min. Total Akt and ERK 1/2 was used as loading control. Western blot analysis is performed on whole cell lysate from VECs in CM miR193a/CM mimic CTR. Experiments were performed at least 3 times in triplicates or quadruplicates and data are represented as mean ± SD. *p* < 0.0001 **** compared to the respective control.

To explore the potential molecular mechanisms underlying the VEC growth inhibitory effects of CM from miR193a-3p transfected MCF-7 cells, we assessed its impact on key signal transduction enzymes ERK1/2 and PI3K-Akt. Abnormal activation of both pathways play a critical role in cell growth associated with BC progression and metastasis and serve as a therapeutic target in many cancers including BC [38,39]. Hence, we performed Western Blot analysis to evaluate Akt phosphorylation by measuring p-Akt levels in VECs exposed to CM from MCF-7 cells transfected with miR mimic control and miR193a-3p. Interestingly, in contrast to our previous observation that CM from MCF-7 induces VEC growth by activating PI3K-Akt [11], CM from miR193a-3p transfected MCF-7 cells inhibited Akt phosphorylation in VECs. As compared to CM from mimic control transfected MCF-7 cells, Akt activation was inhibited by 69.5% ± 10.3 in VECs exposed to CM from miR193a-3p transfected MCF-7 cells (Figure 6a).

ERK1/2 or MAPK pathway comprises of several phosphorylation events that play important role in tumorigenesis as it regulates cell proliferation, apoptosis and migration. Abnormal activation of MAPK pathway is often reported in human cancer, including BC [40]. Hence, we investigated the impact of CM from miR-193a-3p transfected MCF-7 cells ERK1/2 phosphorylation in VECs. In contrast to our recent findings that MCF-7 secretome induces VEC growth by upregulating ERK1/2 phosphorylation [11], CM from MCF-7 cells pre-transfected with miR193a-3p dramatically inhibited ERK1/2 phosphorylation (Figure 6b). Compared to VECs exposed to CM from MCF-7 cells transfected with mimic control, CM from miR193a-3p transfected MCF-7 cells downregulated ERK1/2 phosphorylation in VECs by 46% ± 16.8 (Figure 6b).

### 3.6. Microarray Analysis

#### 3.6.1. Differentially Regulated Genes

**Figure 7:**
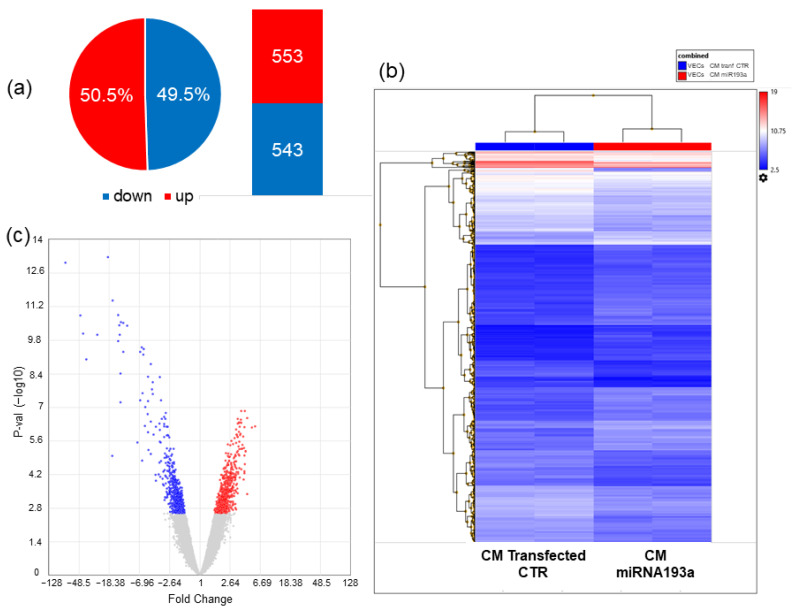
Differentially regulated genes (DRGs) in VECs cultured in CM from MCF-7 cells pre-transfected with miR193a-3p or Transfected CTR (mimic CTR). Figure depicting number of DRGs and pie chart representation of up- and down-regulated genes as percent (%) of total number of DRGs (**a**). Heatmap representation of DRGs between CM miR193a vs CM from transfected CTR (**b**). Volcano plot showing the most up-regulated genes, the most down-regulated genes (green), and the most statistically significant genes are towards the top (**c**). Transcriptome Analysis Console (TAC, Applied Biosystems) was used for analyzing gene expression data.

In order to identify VEC genes regulated by CM from miR193a3p transfected MCF-7 cells (CM miR193a), we performed microarray analysis. Using a cut off *p*-value < 0.05 and 1.5 fold change (FDR) we identified 1096 differentially regulated genes (DRGs) in VECs cultured in CM miR193a compared to VECs cultured in mimic control CM. Furthermore, the volcano plot showed that of all the DRGs, 553 genes were up-regulated and 543 genes were down-regulated (Figure 7).

The top ten genes up-and downregulated are listed in Table 1 and Table 2 respectively.

#### 3.6.2. Validation of Differentially Regulated Genes by Quantitative Real Time PCR

**Figure 8:**
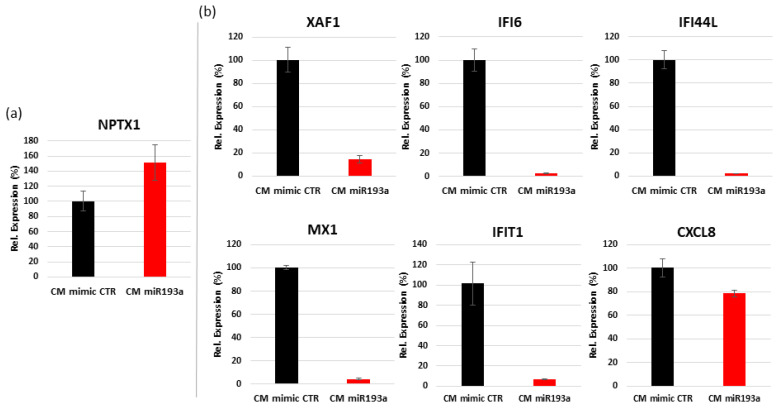
Validation of differentially regulated genes by RT2-PCR in VECs. Seven differentially regulated genes were randomly chosen, and validated by RT-PCR using a custom-designed RT2 PCR array from Qiagen. NPTX1 up-regulated (**a**), XAF1, IFI6, IFI44L, MX1, IFIT1 and CXCL8 downregulated (**b**). GAPDH and PDGFRB were used as internal controls and data normalization. The experiment was performed once in triplicates.

Some differentially regulated genes were randomly chosen, and validated by quantitative RT-PCR. The upregulated gene NPTX1 (−log2 FC 0.93) showed increased expression in PCR (Figure 8a) and the downregulated expression profile of the genes XAF1 (−log2 FC −4.07), IFI6 (−log2 FC −5.41), IFI44L (−log2 FC −5.53), MX1 (−log2 FC −5.26), IFIT1 (−log2 FC −6.24) and CXCL8 (−log2 FC −0.66) showed a potential reduced expression in PCR (Figure 8b). These results were consistent with the microarray genes log fold change.

#### 3.6.3. Pathway Enrichment Analysis of DRGs

GO term enrichment analysis, including biological process was used to determine the biological significance of DRGs in VECs exposed to CM miR193a vs. CM mimic control. The enriched DRGs were mainly in interferon signaling pathways, cellular response to type I interferon, interferon-γ, interferon-β, and interferon-α. Additionally, antigen processing of exogenous peptide antigen via MHC class I TAP dependent/independent signaling pathways were also regulated (Table 3). Comparing pathways enrichment analysis from GO biological process and Bioplanet pathways we found overlap between the interferon signaling pathways. Supplemental Table S1 provides the full list of changes using BioPlanet, GO Biological Process, KEGG, GO Cellular Component, GO Molecular function and MSigDB Hallmark.

#### 3.6.4. Gene’s Identification in Biological Pathways

As shown above, up- and down-regulated genes in response to CM miRNA culture were especially involved in interferon signaling pathways including several genes such as members of the Interferon-induced transmembrane family (IFITM1, IFITM2, IFITM3), interferon-induced protein with tetratricopeptide repeats genes (IFIT1, IFIT2, IFIT3, IFIT5), Interferon alpha inducible proteins (IFI27, IFI6), Interferon-induced GTP-binding protein MX (MX1, MX2), human leukocyte antigen system (HLA-A, HLA-B, HLA-C, HLA-F), 2′-5′-oligoadenylate synthetase (OAS1, OAS2, OAS3), proteasome subunit alfa/beta and proteasome inhibitor (PSMA3, PSMB8, PSMB9, PSMF1). Additionally, ubiquitin proteins (UBA7, UBE2L6, USP18, ISG15), some chemokines (CCL20, CCL23, CCR7, CCL10) and other proteins (ADAR, IFNB1, STAT1, ISG15, XAF1, IRF9) were also involved in the interferon response (Table 4).

### 3.7. Angiogenesis Proteome Profiler in VECs

**Figure 9:**
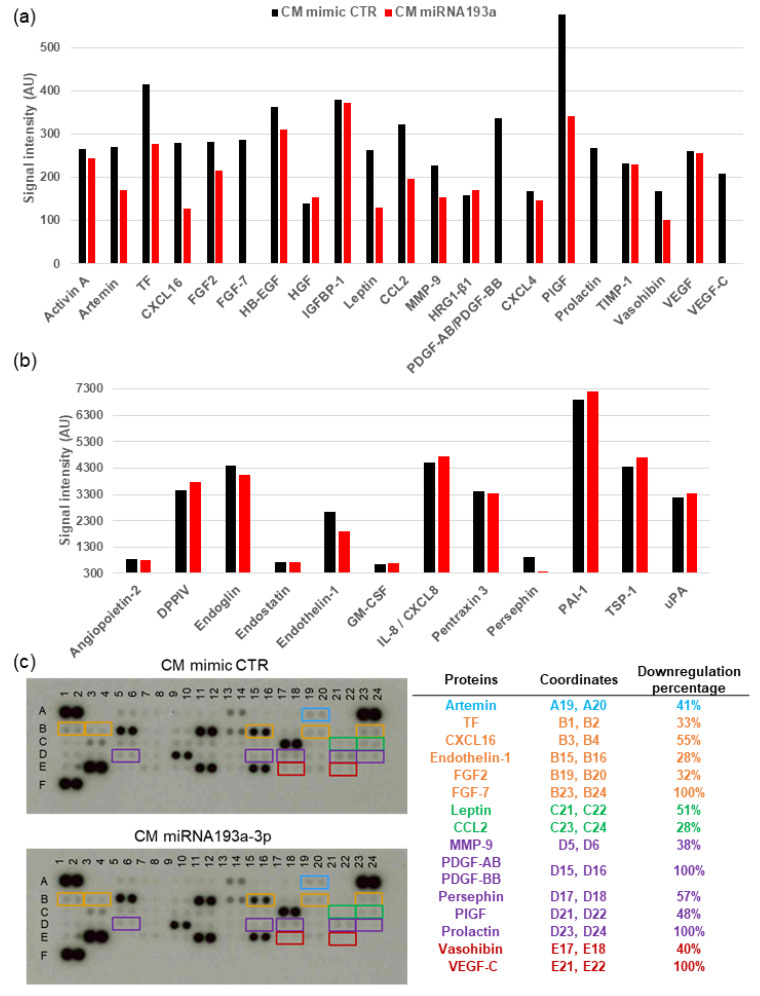
Secretome from miR193a transfected MCF-7 cells inhibits expression of pro-angiogenic proteins in VECs. Angiogenic protein profile array was performed using 200 µg of protein from VECs cultured for 48 h in CM miRNA. Images were analyzed using ImageJ after background subtraction. Bar graphs show the average signal intensities of the framed spots on the array blots and include expression levels changes between 0 and 600 AU (**a**) and between 300 and 7300 AU (**b**). Representative array blots after an exposure time of 5 min (**c**). The table on the right of the array blots lists the most down-regulated proteins and the coordinates for their color marked location on the blots.

Since CM miR193a-3p inhibited proliferation in VECs, we hypothesized that secretome from miR193a-3p transfected MCF-7 cells would be altered and contain secreted factors that inhibit VEC growth and angiogenic activity. To test this hypothesis, we used a Proteome Profiler Antibody Arrays Kit for Human Angiogenesis to determine changes in 55 angiogenesis-related factors in VECs in response to miR193a-3p CM. To quantify changes in protein expression, we analyzed the means of the pixel number of the pair of duplicate spots and plotted the changes of signal intensity in protein levels (5 min exposure time). We found that 20 angiogenesis-promoting proteins were downregulated in VECs by miR193a-3p CM. As depicted in Figure 9, the proteins downregulated by 5–20% were activin A, angiopoietin-2, endoglin, endostatin, HB-EGF, platelet factor 4; by 20–50% were artemin, coagulation factor III/TF, endothelin-1, FGF basic/FGF2, MMP-9, PIGF, vasohibin; by 50–90% were CXCL16, leptin, persephin; and by 100% were FGF-7/KGF, PDGF-AB/PDGF-BB, VEGF-C, and prolactin. These results suggest that miR193a-3p CM inhibits VEC growth by downregulating proangiogenic proteins. Our findings imply that miR193a-3p can potentially alter the balance of angiogenic proteins in the TME by modulating their expression. Importantly miR193a-3p CM mainly down regulates critical proangiogenic proteins in VECs.

### 3.8. miR193a-3p Abrogates E2 Induced Growth in VECs and MCF-7 Cells

**Figure 10:**
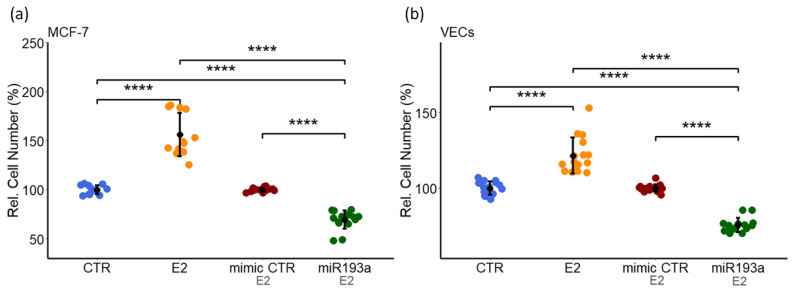
miRNA193a-3p abrogates Estradiol (E2) induced proliferation of MCF-7 and VECs. Cells transfected with or without miR mimic control (mimic CTR) or miR193a-3p (miR193a) were treated with 10 nM E2 or its vehicle (DMSO) as control (CTR). Cell proliferation assessed by counting of MCF-7 (**a**) and VECs (**b**) after 3 days of treatment. Data represent the mean ± SD of at least three independent experiments in triplicates or quadruplicates. *p* < 0.0001 **** compared to the respective control.

Estradiol (E2) is known to stimulate growth of both MCF-7 (ER-positive BC epithelial cells) and VECs [30,34] and is associated with BC/tumor growth and progression [34]. Since miRNAs are important post-transcriptional regulators of genes involved in cell growth and miR-193a-3p has anti-tumor actions [38], we investigated if it can block E2 induced growth of MCF-7 cells and VECs. Treatment of MCF-7 and VECs with 10 nM of E2 for 3 days induced cell proliferation (Figure 10). The growth stimulatory effects of E2 in both MCF-7 and VECs, were abrogated and reversed in cells transfected with miR193a-3p. As compared to MCF-7 and VECs treated with mimic control miR, a 31% ± 9.5 and 25% ± 4.7 inhibition was observed in miR193a-3p transfected MCF-7 and VECs, respectively (Figure 10). Moreover compared to control and E2 treatment, growth of MCF-7 and VEC in response to E2 was significantly downregulated in miR193a transfected cells suggesting that miR193a significantly abrogates E2 induced proliferation of MCF-7 and VECs. 

### 3.9. Estradiol (E2) Decreases miRNA193a-3p Expression in MCF-7 and VECs

**Figure 11:**
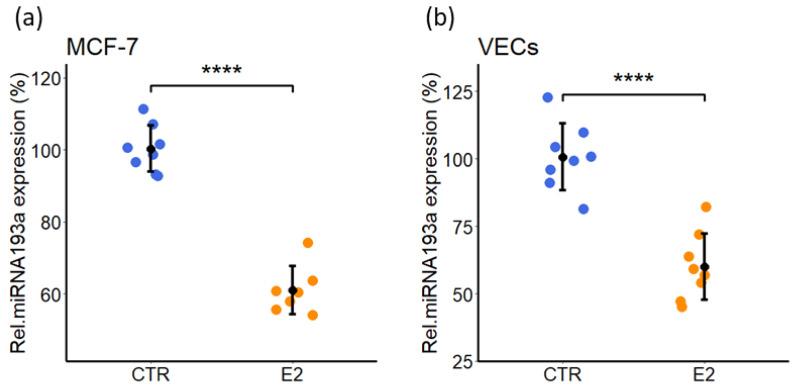
Estradiol (E2) downregulates miRNA193a-3p expression in MCF-7 and VECs. Starved cells (MCF-7 or VECs) were treated for 72 h with 10 nM E2. Subsequently, total RNA was extracted and relative miRNA193a expression levels quantified by RT-PCR using TaqMan miRNA assay. E2 significantly downregulates miRNA193a expression in MCF-7 cells (**a**) and VECs (**b**). The results were normalized to U48 and U49. Results represent mean ± SD; n = 3. *p* < 0.001 **** compared to the respective control.

Based on our observations that miR193a-3p abrogates E2 induced growth in MCF-7 cells and VECs, we assessed whether the growth promoting effects of E2 are in part, mediated via downregulation of endogenous miR193a-3p. We analyzed the effects of E2 on miR193a expression in both MCF-7 and VECs. Cells starved with steroid free medium were treated with 10 nM E2 for 72 h after which RNA was extracted. MiR193a expression, was subsequently analyzed by RT-PCR. Treatment with E2 reduced expression of miR193a by 40% ± 6.67 and 40% ± 12.4 in MCF-7 cells and VECs, respectively (Figure 11). This finding suggests that E2 may induce MCF-7 and VEC growth, a key aspect of BC progression, by downregulating endogenous miR193a-3p expression.

### 3.10. Growth Effects of E2 on MCF-7 and MCF-7 Plus VEC Spheroids

**Figure 12:**
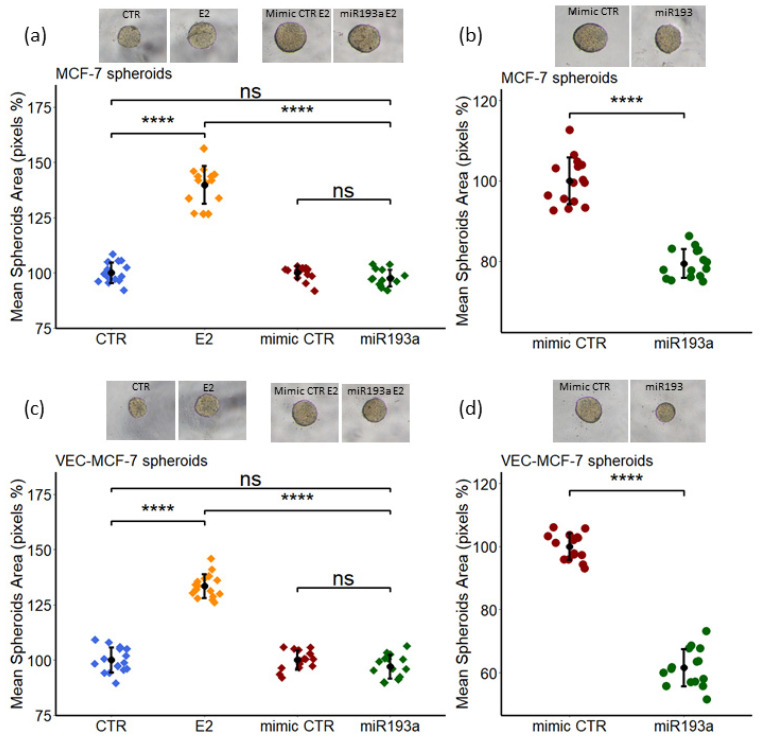
E2 and FCS induced spheroid (MCF-7 and MCF-7+VEC) growth is abrogated by miRNA193a-3p. Pixel analysis was performed to calculate total area of spheroids formed with MCF-7 cells alone (**a**,**b**) or in combination with VECs (**c**,**d**). Cells were transfected with 25 nM of mimic CTR and miRNA193a using Lipofectamine2000 and seeded in U-bottom plate and fed treatment medium containing 10 nM E2 or 2.5% FCS. Pictures of spheroids were taken on Day 4 and analyzed using ImageJ software to calculate spheroid area. *p* < 0.0001 **** compared to the respective control. Data represent the mean ± SD of 15 spheroids. ns = not significant.

Increasing evidence suggests that cells in 3D co-culture systems represent a cellular organization similar to the primary tissue, in vivo. Here, we employed 3D spheroid models to investigate the effects of miR193a-3p on the growth of MCF-7 and MCF-7 plus VEC spheroids. Moreover, we assessed the effects of E2 on the growth of spheroids, which mimic tumor like biology. Cells (MCF-7 or MCF7 + VEC 50:50 ratio) were seeded in 96 well U-bottom plates. Spheroids formed after 48 h, were cultured in medium containing E2. After 4 days in culture, spheroid growth in response to E2 was assessed by microscopically measuring change in total spheroid area (Figure 12). Image analysis of spheroids showed that as compared to vehicle treated control, E2 significantly increases spheroid growth/area in both MCF-7 (by 40% ± 8.5) and MCF-7+VEC (by 33% ± 5.33) spheroids (Figure 12).

We also assessed whether miR193a-3p modulates E2 and FCS induced growth of MCF-7 and MCF-7+VEC spheroids. To accomplish this, cells (MCF-7 or MCF-7 plus VEC) grown in 2D cultures were transfected using Lipofectamine2000. After 24 h, the cells were trypsinized, counted and seeded in U-bottom plates to form spheroids. The spheroids were treated with E2 for 4 days in 3D culture. Image analysis of spheroids showed that the transfection with miR193a-3p abrogates the stimulatory effects of E2 (Figure 12) on the growth of MCF-7 and MCF-7+VEC spheroids.

### 3.11. Inhibitory Effects of miR193a-3p on E2 and FCS Induced Growth of Spheroids

To evaluate whether miR193a-3p affects the growth of MCF-7 and MCF-7+VEC spheroids, both cell types (MCF-7 and VEC) were transfected using Lipofectamine2000 in 2D cultures. Then, the cells were trypsinized, seeded in U-bottom plates to form spheroids and allowed to grow for 4 days in growth medium containing 2.5% steroid free fetal calf serum. Image analysis of spheroids showed a significant growth inhibition of spheroids transfected with miR193a-3p. Compared to mimic control, miR193a-3p inhibited growth of MCF-7 and MCF-7+VEC spheroids by 21% ± 3.6 and 38.5% ± 6, respectively (Figure 12b,d). These results demonstrated that miR193a-3p has inhibitory effects of spheroids growth.

### 3.12. Immunohistochemistry

Immunohistochemical staining was performed to assess cell proliferation in spheroids formed by MCF-7 cells and VECs transfected with miR193a-3p. Histological sections were stained with H&E (hematoxylin and eosin) to examine spheroid architecture and Ki67 antibodies to assess cell proliferation.

**Figure 13:**
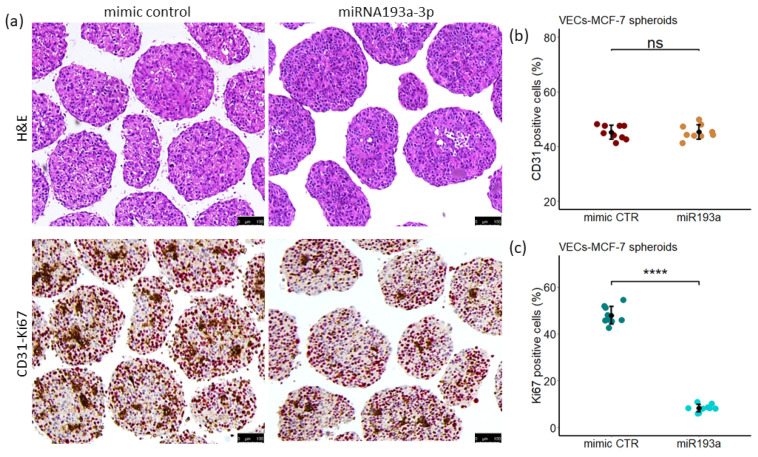
Immuno-histochemical staining of spheroids transfected with mimic control and miR193a-3p. (**a**) Upper panels depict MCF-7+VEC transfected spheroid sections stained for H&E to highlight spheroid structure. Lower panels depict double IHC staining and bright field images of mimic control and miR193a transfected spheroids stained with CD31 an endothelial cell marker (red) and Ki67 as proliferative marker (brown). This image shows the distribution of VECs (stained) and MCF-7 (unstained) cells in the spheroid, as well as the proliferation activity in mimic control and miR193a treated spheroids. CD31 (**b**) and Ki67 (**c**) positive cells were calculated in MCF-7+VEC spheroids transfected with miRNA using ImageJ software. *p* < 0.0005 ****. ns = not significant. The percentages were calculated compared to the total number of cells within the spheroids. Scale bar, 100 μm. Images from at least 10 spheroids were compared for qualitative and semi-quantitative analysis.

**Figure 14:**
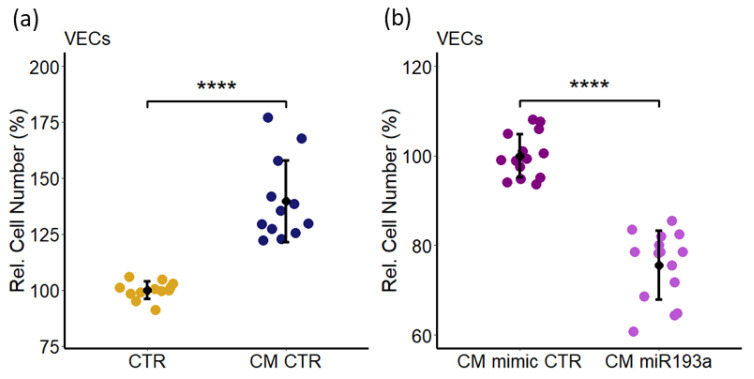
MDA-MB-231 secretome induced growth of VECs are lost in secretome from miR193a-3p transfected cells. VECs were cultured in CM for 48 h and cell proliferation was assessed by cell counting. (**a**) Stimulatory effects of MDA-MB231 CM compared to control CM collected under identical conditions from flaks devoid of cells. (**b**) Effects of CM miR193a produced by MDA-MB231 cells pre-transfected with miRNA193a or mimic CTR. Cell proliferation was reversed from a stimulatory effect of + 40% in CM CTR to minus 25% in VECs treated with CM miR193a. Experiments were performed at least 3 times in triplicates and the results are presented as mean ± SD. *p* < 0.00001 **** compared to the respective control. The experiments were conducted simultaneously and represents continuation of our previous work.

H&E stained spheroids showed healthy, regular and compact structures. No obvious abnormalities were observed in spheroids made from MCF-7+VEC transfected with mimic control and miR193a-3p (Figure 13a). Double immunostaining of spheroid sections revealed the distribution of endothelial cells (stained with CD31 antibody, red) and the proliferating cells within the spheroid (stained with Ki67 antibody, brown; Figure 13a). Quantitative image analysis of stained cells within the MCF-7+VEC spheroid estimated that 55% of the cells were epithelial cells (MCF-7) and 45% were endothelial cell. Importantly, the population of Ki67 positive, proliferating cells was 48% ± 4 in mimic control spheroids and 8.3% ± 1.6 in miR193a-3p spheroids and the population of CD31 positive, endothelial cells was 45% ± 2.6 in both mimic control and miR193a-3p spheroids (Figure 14b,c). The inhibitory actions of miR193a-3p are clearly visible in magnified spheroid section stained with Ki67 (Supplementary File Figure S2).

### 3.13. MDA-MB-231 Secretome Induced VEC Proliferation are Lost in Secretome from miR193a-3p Transfected Cells

To assess whether similar to MCF-7 cells, secretome from other BC cells also induce VEC growth, we investigated the effects of secretome from MDA-MB-231 BC cells. As shown in Figure 14a, treatment of VECs with MDA-MB-231 CM significantly induced VEC proliferation (≈ 40%). Moreover, CM from miR-193a-3p transfected MDA-MB-231 failed in induce VEC growth and inhibited cell proliferation by 25%± 4.8 (Figure 14b). These findings suggest that proangiogenic actions of BC cell secretome are present in both E2 responsive and E2 independent BC cells and may play an important role in most BC tumor angiogenesis. Importantly, similar to MCF-7 cells, miR-193a-3p abrogates BC induced VEC growth by altering MDA-MB-231 secretome, suggesting that miR193a-3p blocks production of key proangiogenic factor(s) by characteristically different BC cells.

## 4. Discussion

Angiogenesis actively contributes to the growth and progression of solid cancers, including BC [7,8,9]. BC cell secretome in the tumor microenvironment (TME) facilitates neo-angiogenesis by promoting VEC growth. Drugs that block BC cell growth or angiogenesis that can restrict tumor growth are of therapeutic relevance. Moreover, molecules that target both BC and VEC growth and BC secretome may be more effective in treating BC. Since small, non-coding microRNAs (miRs) regulate cell growth and miR193a-3p has onco-suppressor activity, we investigated whether miR193a-3p inhibits MCF-7 driven growth (proliferation, migration, capillary formation) of VECs and the underlying signal transduction. Since estrogen is linked to ER positive BC, we also assessed the impact of miR193a-3p on estradiol (E2)-induced growth of MCF-7 cells and VECs. Using MCF-7 cells and VECs grown in monolayers or 3D spheroids and gene microarrays, we provide the first evidence that pro-growth effects of MCF-7 secretome/conditioned medium (CM) are lost in CM collected from MCF-7 cells pre-transfected with miR193a-3p. Moreover, miR193a-3p-CM inhibits MAPK and Akt phosphorylation in VECs. In gene-expression studies, miR193a-3p-CM upregulated 553 genes and downregulated 543 genes in VECs. Differentially regulated gene and pathway analysis highlighted that miR193a-3p CM downregulates interferon-associated genes and pathways implicated in BC/tumor growth and angiogenesis. Moreover, angiogenesis proteome array confirmed the downregulation of 20 pro-angiogenesis proteins by miR193a-CM in VECs. In MCF-7 cells and VECs, estradiol (E2) induced growth and downregulated miR193a-3p expression. Moreover, ectopic expression of miR193a-3p abrogated the growth stimulatory effects of E2 and serum in MCF-7 cells and ECs, as well as in MCF-7 and MCF-7+VEC 3D spheroids. Immunostaining of MCF-7+VEC spheroid sections with ki67 showed miR193a-3p inhibits cell proliferation. Taken together, our findings provide first evidence that miR193a-3p abrogates MCF-7-driven growth of VECs by altering MCF-7 secretome and downregulating pro-growth interferon signals and multiple proangiogenic proteins. Moreover, miR193a-3p can directly inhibit serum and E2 induced growth of MCF-7, VECs, and MCF-7+VEC spheroids. Our findings provide the first evidence that miRNA193a-3p can potentially target/inhibit BC tumor angiogenesis via dual mechanism: altering proangiogenic BC secretome/TME and inhibiting VEC growth. It is feasible that miR193a-3p may target BC growth via a three-pronged manner by inhibiting MCF-7 and VEC growth and by altering MCF-secretome to abrogate its mitogenic and angiogenic actions in VECs. Importantly, miR-193a-3p may inhibit BC growth by potentially modifying TME to abrogate angiogenesis and cancer cell proliferation. It may represent a therapeutic molecule to target breast tumor growth.

Angiogenesis actively contributes to the growth and progression of solid cancers, including BC [3,4,6]. Since cross talk between BC cells and VECs triggers pro-growth molecular mechanisms in both BC cells and VECs [3,6], molecules that can block this pro-growth cross talk may be of therapeutic relevance in treating BC. MicroRNAs regulate cell function [12] and represent a new class of molecules with therapeutic potential. Together with the fact that miR193a-3p is a tumor-suppressor miR [33], which is downregulated in BC [31], we hypothesize that miR193a-3p is a negative regulator of BC and may inhibit BC progression by targeting BC cells, VECs, and inhibiting MCF-7-driven growth of VECs [11].

Our finding that miR193a-3p abrogates VEC growth by altering MCF-7 secretome suggests that it may influence the tumor microenvironment (TME), which plays a key role in driving tumor growth and progression. The breast TME is composed of active molecules generated by multiple cells within its architecture, including cancer cells, fibroblasts/stromal cells, vascular and lymphatic endothelial cells, pericytes, and lymphocytes/immune cells [41]. Importantly, soluble factors and other non-cellular components play a dynamic role in influencing growth of both cancer and VECs [11]. Although, communication between different cells within the TME plays an important role, the cross talk between BC cells and VECs is of particular relevance. Tumor neo-angiogeneis supports tumor tissue perfusion and provides nutrition to support growth. Moreover, EC-derived factors can promote BC cell growth in a paracrine fashion [3,4,6]. Interestingly, MCF-7 secretome also induces proliferation, migration, and capillary formation in VECs, but not in lymphatic ECs [11]. Moreover, LEC-derived factors educate BC cells to produce more pro-growth molecules [42] in their secretome to enhance tumor growth, accompanied by increased angiogenesis [42]. The above findings, together with our observation that miR193a-3p alters MCF-7 secretome to abrogate its growth-promoting actions in VECs, suggests that miR193a-3p may mediate its anti-tumor actions by modulating MCF-7 secretome and be of therapeutic potential.

Abnormal growth of BC cells and vascular neo-angiogenesis in tumors is a hallmark for BC growth/progression [8,9] and a key target to treat BC. Molecules that target their growth are of therapeutic/clinical relevance. It is well-established that targeting cancer/BC cells alone is not always effective due to cancer cell mutations [3], whereas molecules’ targeting of angiogenesis failed in preventing BC patient survival [10]. The above findings, together with the fact that multiple cell types and factors in the TME drive tumor growth [3], indicate that there is growing consensus that a multi-pronged approach of targeting multiple cells or factors that promote BC growth may be more effective in treating BC. Our finding that miR193a-3p alters MCF-7 secretome from pro- to anti-growth and inhibits the growth of MCF-7 and VECs suggests that it may inhibit BC growth via a multipronged mechanism. Since, in addition to MCF-7 and VECs, stromal/fibroblasts and other cells may also contribute to BC growth, further studies to assess the impact of miR193a-3p on key growth-promoting cells in BC tumors is required. A better understanding of the cross-talk mechanism(s) involved in driving BC growth would help in developing molecules, which can target the BC microenvironment in a multipronged manner.

Recent studies provide evidence that biologically active microRNAs have therapeutic potential [20]. Multiple miRNAs play a growth modulatory role in BC [13]. Since miR193a-3p induces onco-suppressive actions in multiple types of cancer [38] and its expression is reduced in BC, it may be effective in inhibiting BC growth. The fact that miR193a-3p targets cyclin D1, a key pro-growth cell cycle regulating protein in both cells, it is feasible that it induces anti-mitogenesis in both BC cells and VECs. Since estrogen induces growth in MCF-7 (ER-positive BC cells) as well as in VECs, it is feasible that miR193a-3p is effective in targeting growth of both BC cancer cells and VECs. Consistent with the above notion, in the present study, miR193a-3p inhibited growth in both MCF-7 and VECs.

Angiogenesis is a critical process in tumor progression. The fact that CM acts as a major factor in modulating EC migration, proliferation, and capillary formation suggests BC cell secretome may play a critical role in the pathophysiology of BC tumors. In the present study, CM collected from MCF-7 cells pre-transfected with miR193a-3p inhibited VEC growth and capillary formation. This observation, together with the fact that other cells within the TME (e.g., lymphatic ECs) educate BC cells to produce more pro-growth secretome [42], suggests that miR193a-3p may alter BC cell/MCF-7 secretome to abrogate/dilute the mitogenic actions of paracrine factors generated by BC cells, thereby inhibiting VEC proliferation and angiogenesis. Since BC cell secretome plays a key role in promoting angiogenesis, the modulatory effects of miR193a-3p may be of critical importance in limiting BC tumor growth/progression. This contention is supported by our observation that miR-193a-3p CM downregulated 20 proteins known to induce angiogenesis. Although promising, this contention needs to be further tested and confirmed in an in vivo setting.

Apart from proliferation, migration plays a key role in tissue infiltration by vascular endothelial cells to facilitate new vessel formation. Moreover, migration of BC cells contributes to metastasis. In this context, Xie and colleagues [33] have found that miR193a-3p overexpression inhibited the migration of BC cells. We have recently shown that MCF-7 secretome induces VEC migration and capillary formation [11]. Whether miR193a-3p modulates the generation of paracrine factors in MCF-7 secretome remains unknown and was tested. In VECs exposed to MCF-7 CM from miR193a-3p-transfected cells, we observed delayed wound closure and anti-migratory effects, suggesting that miRNA193a-3p exposure alters MCF-7 secretome and reduces its pro-migratory activity on VECs. These findings suggest that miR-193a-3p may modulate the TME and prevent VEC capillary infiltration. Since cell migration is also involved in metastasis, miR193a-3p-induced changes in secretome may also prevent the spread of cancer cells and metastasis. However, further in-depth studies are required to confirm this notion.

The PI3K/AKT [39] and MAPK/ERK1/2 [37] pathways are involved in cancer development and metastatic progression. Moreover, inhibiting Akt and ERK activity prevents tumor growth and metastasis [40]. Our finding that secretome from miR193a-3p-treated MCF-7 cells inhibited ERK1/2 and Akt phosphorylation suggests that miR193a-3p prevents the release of ERK and Akt activating proangiogenic factors from BC cells. Since the miRNA levels were undetectable in the secretome, the potential role of leached miR193a-3p in MCF-7 secretome can be ruled out. Since inhibitors of both ERK1/2 and Akt are of clinical relevance to treat BC progression [43,44], miR193a-3p may also represent a promising candidate due to its dual inhibitory actions.

To identify the molecular mechanisms influenced by the MCF-7 secretome, we used a microarray approach and investigated the transcriptome profile of VECs exposed to miR193a-CM. In DRG analysis, we identified 553 upregulated and 543 downregulated genes between VECs cultured in CM miR193a and its relative control (CM mimic CTR). According to the functional annotation results of the data, the DRGs were mainly involved in defense response, viral processes, cell proliferation, viability, differentiation, and apoptosis. In order to understand the interaction of the DRGs, we performed GO Biological process and BioPlanet pathways analysis. It highlighted that DRGs that were significantly enriched represented type I and II interferon signaling pathways. Since many DRGs participate in immune response, tumor growth inhibition, and apoptosis [45,46,47], it is feasible that miR-193aCM inhibits VEC growth via interferon signaling mechanism(s).

The most significant enriched pathways were interferon alpha/beta signaling (Bio-Planet, adjusted *p*-value 1.74 × 10^−12^) and cellular response to type I interferon (GO: 0071357, adjusted *p*-value 1.93 × 10^−16^). Type I interferon, including IFN-α and IFN-β, has contradictory effects; it directly promotes antitumor response, suppressing tumor growth and induces tumor progression by establishing conditions in the tumor microenvironment that facilitate tumor growth [48,49]. The DRGs of interferon alpha/beta signaling and cellular response to type I interferon that were downregulated include genes involved in tumor progression (CCL20 [50], SAMHD1 [51], TRIM 22 [52], PLSCR1 [53], IFITs (IFIT1, IFIT2, IFIT3, IFIT5) [54], PML [55], PSMF1 [56], TAPs (TAP1, TAP2) [57], LGALS9 [58], MX1 [59]), cell proliferation, metastasis, angiogenesis (TRIM 38 [60], MX2 [61], PSMA3 [62], PSMB8 [63], PSMB9 [64], CXCL10 [65], IFI27 [66], IRF9 [67], OASs (OAS1, OAS2, OAS3) [68], IFITMs (IFITM1, IFITM2, IFITM3) [69], UBE2L6 [70], USP18 [71]), tumorigenesis, and genes that play roles in cancer (STAT1 [72], TRIM38 [60], NUP38 [73], RSAD2 [74], IFI6 [75], HLAs (HLA-A, HLA-B, HLA-C, HLA-F) [76], XAF 1 [77], ADAR [78]). The up-regulated genes were involved in apoptosis, cell-death, cell growth inhibition (PID1 [79], TRIM31 [80], CCL23 [81], CCR7 [82], EIF4A2 [83], GAS6-AS1 [84], UBE1L (UBA7) [85]). The present analysis suggests that many genes specific for apoptosis, cell proliferation, and migration processes show high changes in expression in vitro culture of VECs in miR193a-CM.

In VECs treated with CM from miR193a-3p-transfected MCF-7 cells, the magnitude of change for the top 10 downregulated genes (3.37–6.24 fold) was much higher than that of upregulated genes (2.1–2.58 fold), suggesting that the impact of downregulation may be of greater significance in modulating VEC function. Many of the upregulated genes, i.e., PARP8, ATP6, V1G3, NANOGNB, and CALB1, have been reported to be upregulated in various cancer cells. Since CM derived from miR193a-3p-transfected MCF-7 cells inhibited both proliferation and migration in VECs and LECs, these pro-growth genes may have roles other than growth regulation. For example, PARP8 has been shown to change cell structure and phenotype [86]. Within the downregulated genes, the most prominent group were interferon-regulated genes, i.e., IFIT1, IFITM1, IFIT3, and IFI44L. Although a long-standing view is that interferon inhibits both angiogenesis and cancer cell growth, more recent studies provide evidence that it also mediates pro-tumorigenic effects [54]. Indeed, studies involving IFIT protein (IFIT1, IFIT3) functions and their participation in various molecular signaling mechanisms implicates them in cancer progression and metastasis [87]. Interestingly, the pro-cancer growth effects of IFIT proteins involve Akt-phosphorylation, which was also downregulated in VECs exposed to CM from miR193a-transfected MCF-7 cells. This suggests that the autocrine factors in CM inhibit growth, potentially downregulating IFIT proteins and inhibiting Akt [87]. Interestingly, IFITM1 overexpression has been shown to enhance the aggressive phenotype of SUM149 inflammatory breast cancer cells in a signal transducer and activator of transcription 2 (STAT2)-dependent manner [88]. Moreover, interferon-stimulated genes are repressed by progesterone, which counteracts the growth-inducing effects of estrogen in BC [89]. Taken together, our finding that the interferon-associated proteins and signaling are downregulated suggest that miR193a-3p facilitates the release of autocrine factors from MCF-7 cells that prevent angiogenesis by downregulating specifically the pro-growth actions of interferons.

Our finding that CM from miR193a-3p-transfected MCF-7 cells strongly downregulated interferon-associated genes in VECs was unexpected. The fact that miR193a-3p CM inhibits VEC growth (and that this is associated with downregulated multiple interferon-induced transmembrane protein genes, including IFITM1, IFIT1, IFIT3 and interferon pathways) suggests they may play a prominent role in mediating growth inhibitory actions in VECs. Studies from Hughes group [90,91] provide evidence that IFITM1 is highly expressed in ECs during sprouting and lumen formation, processes which are disrupted by knockdown of IFITM1 [90,91]. IFITMs are suggested to regulate the transition of ECs from quiescent to angiogenic state [90,91]. Interestingly IFIT1 and IFIT3 genes are upregulated during cancer metastasis and invasion [54] and mediate their growth-promoting actions by complexing and phosphorylating Hsp90 and its client proteins PKC, EGFR, Akt, and p38 [54], together with our observation that miR193a-3p CM inhibits VEC growth as well as Akt and ERK1/2 phosphorylation, suggests that it may prevent MCF-7-driven angiogenic actions via this mechanism. Indeed, ectopic expression of IFIT1 and IFIT3 has been shown to enhance many EGFR and annexin-2 pathways to induce cell proliferation, survival, and drug resistance [54]. Additionally, we observed downregulation of USP18, which mediates angiogenic effects of IFN-α by promoting EC capillary sprouting and angiogenesis [92,93]. Moreover, USP18 deficiency in mammary cells creates antitumor activity [94]; L-GALS9 which induces angiogenesis and MAPK phosphorylation in VECs [95] and galectins play a critical role in regulating angiogenesis [96]. Taken together, our findings suggest that miR193a-3p may prevent key angiogenic processes, including sprouting and capillary formation by altering the generation of angiogenesis stimulants by BC/MCF-7 cells.

Apart from the interferon-associated mechanism that regulates angiogenesis, CM from miR193a-3p-transfected MCF-7 cells also down regulated MX2, a dynamin-like GTPase and cell cycle regulator, which has been shown to induce growth in a subset of melanoma cells [61]. Moreover, miRNA193a-CM down regulated PARP9, which is shown to be overexpressed in human BC cells and promotes cell migration [97]; as well as DDX60, an interferon-associated gene which is associated with glioma malignancy [98]. Additionally, miR193a-CM upregulated NPTX1, a member of pentraxin family, which inhibits proliferation and promotes apoptosis in cancer cells [99]. Interestingly, the top ten genes up and down regulated in VEC by MCF-7 CM (our recently published manuscript [11]) and miR193a-CM were not the same, suggesting that miR193a-3p alters MCF-7 secretome balance from endothelial growth inducing factors to growth-inhibiting factors are different. Taken together, the above findings suggest that miR193a-3p switches the release of soluble factors from MCF-7 cells from pro- to anti-VEC growth.

Consistent with our findings that MCF-7 CM from miR193a-3p-transfected cells inhibits VEC growth, migration, capillary formation, and downregulates genes and pathways that promote angiogenesis, we also observed downregulation of several pro-growth/proangiogenic proteins in proteome arrays. Treatment of VECs with CM from miR193a-3p pre-transfected MCF-7 downregulated the expression of 20 proteins by 10–100%. The majority of downregulated proteins were those with known proangiogenic effects, such as activin A, angiopoietin-2, artemin, coagulation factor III/TF, CXCL16, Endoglin/CD105, endostatin, endothelin-1, FGF basic/FGF2, FGF7/KGF, HB-EGF, Leptin, MCP-1/CCL2, MMP-9, PDGF-AB/PDGF-BB, Persephin, placental growth factor (PIGF), prolactin, and VEGF-C. However, proteins such as Vasohibin with a dual role in angiogenesis were also downregulated, whereas proteins that negatively regulate angiogenesis (TIMP-1, TSP-1, Serpin 1, and pentraxin 3) remained either unaltered or were marginally (≤3%) upregulated, suggesting that the overall balance is tilted towards anti-angiogenic proteins in VECs treated with CM from miR193a-3p-transfected cells. Our findings suggest that the upregulation of anti-angiogenic proteins by miR193a-3p transfected MCF-7 secretome plays a defining role in mediating its growth inhibitory effects in VECs. In addition to the above-listed antiangiogenic proteins, miR 193a-3p CM also downregulated interferon-associated genes (IFIT1, IFITM1, IFIT3, IFI44L) as well as USP18, OAS2 and LGALS9, which are known to inhibit angiogenesis. We postulate that miR193a-3p inhibits angiogenesis by upregulating multiple antiangiogenic mechanisms. Angiogenesis nourishes tumor growth and plays a crucial role in the spread of cancer cells. Advancing knowledge of the TME and cell–cell interaction in tumors will help improve treatment of cancer patients.

Among the proteins that were downregulated by miR193a-3p CM in VECs and that play a role in the pathophysiology of tumor angiogenesis were: FGF-7/KGF (downregulated by 100%) known to play an important role in tumor angiogenesis [100]; FGF-2 (downregulated by 32%) promotes angiogenesis [101]; HB-EGF (downregulated by 14%) promotes angiogenesis by activating PI3-l kinase and MAPK [102]; PDGF-AB and PDGFBB (downregulated by 100%) family plays a complex but important role in angiogenesis [103]; Persephin (downregulated by 57%) an angiogenesis promoter increased in breast cancer [104]; Prolactin (downregulated by 100%) which is also known as a third hormone in breast cancer pathophysiology [105] and promotes angiogenesis [106]; VEGF-C (downregulated by 100%) induces angiogenesis and influences vascular permeability [107]; Placenta growth factor (PIGF, downregulated by 48%) considered a second member of VEGF family stimulates angiogenesis and is overexpressed in breast cancer [108,109]; Leptin (downregulated by 51%) a key player in energy homeostasis induces angiogenesis [110]; MMP9 (downregulated by 38%) plays an important role in promoting tumor angiogenesis [111]; CCL2 (downregulated by 28%) induces angiogenesis via activation of Ets-1 transcription factor [112]; CXCL16 (downregulated by 55%), a chemokine that stimulates angiogenesis and plays an active role in the pathogenesis of cancer [113,114]; Artemin (downregulated by 41%) promotes angiogenesis in mammary carcinoma by activating TWIST1-VEGF-A signaling [115]; coagulation factor III (TF, tissue factor;downregulated by 33%) a primary initiator of the coagulation cascade that induces angiogenesis and plays an active role in tumor progression [116]; endothelin-1 (downregulated by 28%) an angiogenesis promoter with an important role in tumors [117]. All in all, in VECs, miR193a-CM downregulated multiple proangiogenic proteins that play a key role in angiogenesis. Since miR193a-3p inhibits growth of MCF-7 cells, it is tempting to speculate that factors generated by proliferating and non-proliferating cells may play an important role in defining angiogenesis, and this hypothesis should be further investigated.

MCF-7 cells are ER-positive BC cells which respond to E2. Since E2-induced growth is associated with the pathophysiology of estrogen-sensitive BC, we investigated the role of miR193a-3p in E2-stimulated growth of MCF-7 cells. Our finding that growth-inducing effects of E2 in MCF-7 cells were associated with downregulation of miR193a-3p expression, together with the fact that miR193a-3p inhibits MCF-7 growth, suggests that E2 may induce MCF-7 growth, in part by downregulating miR193a-3p. Apart from ER+ BC cells, E2 also promotes VEC growth and angiogenesis. Hence, we also investigated the role of miR193a-3p in E2-induced growth of VECs. Similar to MCF-7 cells, E2-induced growth of VECs was accompanied with downregulation of miR193a-3p. This observation, together with the fact that miR193a-3p inhibits E2-induced growth in VECs, suggests that miR193a-3p is a negative regulator of MCF-7 and VEC growth and may protect against E2-induced BC growth by targeting both BC cells and angiogenesis. Our contention is further supported by the fact that miR193a-3p levels are decreased in BC [31]. Moreover, estrogen regulates its levels in humans [12]. Apart from E2, multiple other growth factors are known to promote BC growth [3]. Since FCS contains a battery of factors, our observation that miR193a-3p inhibits FCS-induced growth suggests that it may also abrogate BC growth induced by factors other than E2. Moreover, its antigrowth effects may not be limited to ER-positive MCF-7 cells. Indeed, VEC growth was also inhibited by CM from mirR193a-3p-transfected MDA-MB-231 BC cells that are E2-indepenent. However, studies using other BC cells/cell-lines are needed to confirm this notion.

Using 3D spheroids made of MCF-7 and MCF-7 plus VECs, we further confirmed our findings in 2D cultures; 3D spheroids represent a more realistic model to study cell–cell interaction in cancer/tumor [35] and reflect TME. Our finding that miR-transfection does not adversely impact spheroid morphology confirms their integrity. Moreover, dual labeling shows an even distribution of VECs and MCF-7 cells in mixed cell spheroids. Staining with Ki67 provides evidence for proliferating cells in 3D spheroids and that miR193a-3p inhibits proliferating cells, although the specific cell type inhibition could not be inferred. The finding that both FCS- and E2-induced growth of MCF-7 and MCF-7 plus VEC spheroid suggests it is a viable model to assess the role of specific cell–cell interaction in driving cancer growth. Moreover, our finding that the stimulatory effects of FCS as well as E2 were lost in spheroids made from MCF-7 and VECs transfected with miR193a-3p reaffirms that miR193a-3p has inhibitory effects on the growth of both MCF-7 and MCF-7 plus VEC spheroids. Based on these observations, we speculate that miR193a-3p may have inhibitory actions on key cells, which constitute BC TME and participate in driving BC/tumor growth.

Our finding that MCF-7-secretome-stimulated VEC proliferation was mimicked by secretome from MDA-MB-231 cells, which are characteristically different, suggests that BC secretome may play a critical role in driving proangiogenic activity in BC tumors. However, this contention cannot be generalized, as BC cells are known to exhibit differential characteristics and react differently to various treatments and growth stumuli. Since angiogenesis is a hallmark for tumor growth/progression, BC cell secretome may play a key role in defining pro-angiogenic TME. Taken together, our findings provide the first evidence that apart from its direct anti-miotigenic actions in VECs, miR193a-3p abrogates BC cell induced growth of VECs by altering paracrine factors in BC cell secretome. Future studies are required to screen and identify the key growth/angiogenic molecules in the secretome that are downregulated by miR193-3a-3p in BC cells. Moreover, the contribution of significantly modulated DRGs by miR193a-3p in mediating the effects of secretome needs to be investgated. Finally, in vivo studies are required to fully confirm our in vitro observation that miR193a-3p alters BC secretome and inhibits VEC and BC cell growth.

There are several limitations of the present study that should be addressed in future. Although we provide evidence that miR-193a-3p induces antimitogenic actions in VECs, in part by altering MCF-7 secretome, which CM factor(s) were altered and responsible have not been identified. Moreover, the antiangiogenic role of DEGs that are significantly regulated by miR193a-3p secretome in VECs should be further confirmed using silencing and/or overexpression approaches. Moreover, how the selected DEGs are modulated by ERK/Akt and E2 as well as its receptors, particulary ER-α, may help explain their role in regulating BC secretome driven angiogenesis and VEC growth.

## 5. Conclusions

In conclusion, our findings provide the first evidence that miR193a-3p induces anti-mitogenic actions on key components that drive the growth and progression of BC tumors. In this context, miR-193a-3p inhibits growth of VECs and MCF-7 BC cells and abrogates MCF-7-driven growth of VECs by altering its secretome. Our findings suggest that miR193a-3p may target BC growth via a three-pronged mechanism that abrogates key components of BC TME, i.e., BC cell growth, VEC growth, and BC secretome-induced VEC growth. These components play a critical role in BC tumor growth and progression. At a molecular level, miR193a-3p inhibits VEC growth by altering MCF-7 secretome to represses Akt and MAPK phosphorylation and down regulates pro-angiogenic interferon-associated genes and 20 pro-angiogenic proteins in VECs. Since miR193a-3p has a negative growth regulatory role in BC cells and VECs, it may represent a therapeutic molecule to target breast cancer progression. Moreover, circulating levels of miR193a-3p may serve as a potential biomarker in breast cancer. Our observations may provide valuable information to identify paracrine factors altered by miR193a-3p in BC secretome that inhibit VEC growth and may abrogate breast cancer/tumor development. Moreover, the identified genes and proteins could serve as attractive therapeutic targets for counteracting vascular angiogenesis in tumors. Taken together, miR-193a-3p inhibits key components of BC TME (VECs, BC cells, secretome) that drive BC growth and may represent a promising inhibitory molecule against BC.

## Figures and Tables

**Table 1:** Top ten up-regulated genes in VECs cultured in CM miRNA.

Gene Symbol	Description	Log2 FC	FDR *p*-Value
PARP8	poly(ADP-ribose) polymerase family member 8	2.58	0.0002
ATP6V1G3	ATPase H+ transporting V1 subunit G3	2.43	0.0003
NANOGNB	NANOG neighbor homeobox	2.21	0.018
CALB1	calbindin 1	2.2	0.0002
CCDC172	coiled-coil domain containing 172	2.14	0.0015
PCP4	Purkinje cell protein 4	2.12	0.0015
TDO2	tryptophan 2,3-dioxygenase	2.1	8.91 × 10^−5^
AK9	adenylate kinase 9	2.09	0.0034
YEATS2	YEATS domain containing 2	2.08	0.0054
WBP4	WW domain binding protein 4	2.07	0.0002

Transcriptome Analysis Console (TAC, Applied Biosystems) was used to analyze gene expression data of VECs cultured in CM miR193a and CM mimic CTR. The table above shows top ten up-regulated genes along with the respective fold changes (FC) and adjusted *p*-values (FDR *p*-value). For the analysis, we applied a fold change (FC) cut-off of +/− 1.5 and FDR *p*-value of 0.05.

**Table 2:** Top ten down-regulated genes in VECs cultured in CM miRNA.

Gene Symbol	Description	Log2 FC	FDR *p*-Value
IFIT1	interferon-induced protein with tetratricopeptide repeats 1	−6.24	1 × 10^−9^
IFITM1	interferon induced transmembrane protein 1	−4.27	1 × 10^−9^
OAS2	2-5-oligoadenylate synthetase 2	−4.04	2.55 × 10^−8^
IFI44L	interferon-induced protein 44-like	−5.53	6.27 × 10^−8^
IFIT3	interferon-induced protein with tetratricopeptide repeats 3	−3.77	6.27 × 10^−8^
LGALS9	lectin, galactoside-binding, soluble, 9	−3.56	9.37 × 10^−8^
MX2	MX dynamin-like GTPase 2	−3.66	9.37 × 10^−8^
DDX60	DEAD (Asp-Glu-Ala-Asp) box polypeptide 60	−3.37	9.57 × 10^−8^
USP18	ubiquitin specific peptidase 18	−3.73	9.57 × 10^−8^
PARP9	poly(ADP-ribose) polymerase family member 9	−3.71	1.72 × 10^−7^

Transcriptome Analysis Console (TAC, Applied Biosystems) was used for analyzing gene expression data of VECs cultured in CM miR193a and CM mimic CTR. Listed above are the top ten down-regulated genes with the respective fold changes (FC) and adjusted *p*-values (FDR *p*-value). For the analysis, we applied a fold change (FC) cut-off of +/− 1.5 and FDR *p*-value of 0.05.

**Table 3:** Pathway enrichment analysis (Enrichr) of DRGs between VECs cultured in CM miR193a vs. CM mimic CTR.

Pathway	Overlap	Adj. *p*-Value
**BioPlanet**		
Interferon alpha/beta signaling	25/64	1.74 × 10^−12^
Interferon signaling	35/168	4.98 × 10^−9^
Immune system signaling by interferons, interleukins, prolactin, and growth hormones	45/280	4.47 × 10^−8^
Type II interferon signaling (interferon-gamma)	13/50	6.11 × 10^−4^
Antigen processing: cross presentation	15/79	0.005
Endosomal/vacuolar pathway	5/9	0.009
**GO Biological Process**		
cellular response to type I interferon (GO:0071357)	29/65	1.93 × 10^−16^
type I interferon signaling pathway (GO:0060337)	29/65	1.93 × 10^−16^
negative regulation of viral process (GO:0048525)	23/70	1.95 × 10^−9^
defense response to symbiont (GO:0140546)	30/124	4.78 × 10^−9^
defense response to virus (GO:0051607)	31/133	4.85 × 10^−9^
negative regulation of viral genome replication (GO:0045071)	18/54	1.96 × 10^−7^
regulation of viral genome replication (GO:0045069)	19/67	1.21 × 10^−6^
cellular response to interferon-gamma (GO:0071346)	22/121	3.59 × 10^−4^
response to interferon-beta (GO:0035456)	10/28	5.61 × 10^−4^
response to interferon-alpha (GO:0035455)	8/18	8.38 × 10^−4^
interferon-gamma-mediated signaling pathway (GO:0060333)	15/68	0.001
response to cytokine (GO:0034097)	23/150	0.003
antigen processing and presentation of exogenous peptide antigen via MHC class I, TAP-dependent (GO:0002479)	15/73	0.003
antigen processing and presentation of exogenous peptide antigen via MHC class I (GO:0042590)	15/78	0.006
antigen processing and presentation of exogenous peptide antigen via MHC class I, TAP-independent (GO:0002480)	5/8	0.006

Pathway enrichment analysis of DRGs between VECs cultured in CM miR193a and CM mimic CTR. Analysis was performed comparing the BioPlanet and GO Biological Process on the Enrichr website by uploading DRGs obtained by Transcriptome Analysis Console (TAC). In the table are listed the number of regulated genes compared to total number of genes in the pathway (second column), and *p*-value adjusted for multiple testing (last column).

**Table 4:** List of genes involved in the enriched pathways and their potential role.

Gene Symbol	Description	Function	Angiogenesis	Log2 FC	FDR *p*-Value
ADAR	adenosine deaminase, RNA-specific	A to I RNA Editing	Pro	−1.15	0.007
AIF1	allograft inflammatory factor 1	Inflammation	Pro	1.52	0.0326
B2M	beta-2-microglobulin	Immune response	Pro	−1.02	0.0344
BST2	bone marrow stromal cell antigen 2	Immunomodulatory	Pro	−3.68	3.98 × 10^−6^
CCL20	chemokine (C-C motif) ligand 20	Inflammation	Pro	−1.2	0.0348
CCL23	chemokine (C-C motif) ligand 23	Immune response	Pro	1.87	0.0004
CCR7	chemokine (C-C motif) receptor 7	Immune response	Pro	1.19	0.0204
CD47	CD47 molecule	Immune/Integrin	Anti	−1.12	0.0216
CXCL10	chemokine (C-X-C motif) ligand 10	Immunomodulatory	Anti	−2.21	1.53 × 10^−5^
CYBA	cytochrome b-245, alpha polypeptide	Antiviral/Immunomodulatory	Pro	−0.83	0.0297
DDX58	DEAD (Asp-Glu-Ala-Asp) box polypeptide 58	Innate Immunity	Anti	−1.87	0.0002
EIF2AK2	eukaryotic translation initiation factor 2-alpha kinase 2	Innate Immunity	Pro	−2.18	2.21 × 10^−5^
EIF4A2	eukaryotic translation initiation factor 4A2	Cancer growth	NK	−0.73	0.0491
GAS6-AS1	GAS6 antisense RNA 1	Inflammation/Tumor growth	NK	1.03	0.0314
GBP1	guanylate binding protein 1, interferon-inducible	Autonomous Immunity	Anti	−1.45	0.0304
GBP3	guanylate binding protein 3, interferon-inducible	Innate Immunity	NK	1.53	0.0124
HLA-A	major histocompatibility complex, class I, A	Immune defense/anti-viral	Pro	−1.3	0.0036
HLA-B	major histocompatibility complex, class I, B	Immune defense/viral immunity	Pro	−1.65	0.0005
HLA-C	major histocompatibility complex, class I, C	Immune defense/viral immunity	Pro	−1.,63	0.0049
HLA-F	major histocompatibility complex, class I, F	Immune defense/viral immunity	Pro	−1.48	0.0002
IFI27	interferon, alpha-inducible protein 27	Innate Immunity	Pro	−2.89	0.0008
IFI6	interferon, alpha-inducible protein 6	Immunomodulatory/anti-apoptosis/	Pro	−5.41	1.72 × 10^−7^
IFIT1	interferon-induced protein with tetratricopeptide repeats 1	Innate Immunity	Pro	−6.24	1 × 10^−9^
IFIT2	interferon-induced protein with tetratricopeptide repeats 2	Innate Immunity	Anti	−1.26	0.0041
IFIT3	interferon-induced protein with tetratricopeptide repeats 3	Innate Immunity	Pro	−3.77	6.27 × 10^−8^
IFIT5	interferon-induced protein with tetratricopeptide repeats 5	Innate Immunity	Pro	−2.4	5.05 × 10^−6^
IFITM1	interferon induced transmembrane protein 1	Immunomodulatory	Pro	−4.27	1 × 10^−9^
IFITM2	interferon induced transmembrane protein 2	Immunomodulatory	Pro	−1.58	0.0001
IFITM3	interferon induced transmembrane protein 3	Immunomodulatory	Pro	−1.86	5.05 × 10^−6^
IFNB1	interferon, beta 1, fibroblast	Innate Immunity	Anti	−1.04	0.0384
IRF9	interferon regulatory factor 9	Immunity/anti-viral	Anti	−0.96	0.0178
ISG15	ISG15 ubiquitin-like modifier	Anti-viral/Transcription factor	Pro	−2.4	0.0004
LEF1	lymphoid enhancer-binding factor 1	Transcription factor/Wnt signaling	Pro	1.58	0.0023
LGALS9	lectin, galactoside-binding, soluble, 9	Inflammation/Immune response	Pro	−3.56	9.37 × 10^−8^
MAP4K3	mitogen-activated protein kinase kinase kinase kinase 3	Autoimmune/cell stress	Pro	−0.79	0.0331
MME	membrane metallo-endopeptidase	Protease/Metabolism	Anti	1.23	0.0217
MX1	MX dynamin-like GTPase 1	Anti-viral/Immunity	Pro	−5.26	1.10 × 10^−6^
MX2	MX dynamin-like GTPase 2	Innate immunity	Pro	−3.66	9.37 × 10^−8^
NCF1	neutrophil cytosolic factor 1	Innate immunity	Pro	1.27	0.0272
NCF2	neutrophil cytosolic factor 2	Autoimmunity	Pro	1.45	0.0262
NUP88	nucleoporin 88kDa	Nuclear trafficking/oncogenic	Pro	−1.22	0.0172
OAS1	2-5-oligoadenylate synthetase 1	Innate anti-viral	Pro	−4.76	1.72 × 10^−7^
OAS2	2-5-oligoadenylate synthetase 2	Innate anti-viral	Pro	−4.04	2.55 × 10^−8^
OAS3	2-5-oligoadenylate synthetase 3	Innate anti-viral	Pro	−2.54	6.41 × 10^−5^
PID1	phosphotyrosine interaction domain containing 1	Adipocyte growth	NK	0.79	0.0263
PLSCR1	phospholipid scramblase 1	Anti-viral immunity	Pro	−2.61	5.21 × 10^−7^
PML	promyelocytic leukemia	Anti-viral/tumor suppressor	Anti	−1.08	0.0138
PNPT1	polyribonucleotide nucleotidyltransferase 1	RNA metabolism	NK	−1.56	0.0072
PSMA3	proteasome subunit alpha 3	Proteolytic	NK	−1.1	0.0171
PSMB8	proteasome subunit beta 8	Proteolytic/Immunoproteasome	Pro	−1.51	0.0036
PSMB9	proteasome subunit beta 9	Immunoproteasome	Anti	−2.42	0.0001
PSMF1	proteasome inhibitor subunit 1	Immunoproteasome	NK	−0.82	0.0298
RSAD2	radical S-adenosyl methionine domain containing 2	Innate immunity	NK	−2.38	0.0013
SAMHD1	SAM domain and HD domain 1	Anti-viral/tumor suppressor	NK	−2.7	4.9 × 10^−7^
STAT1	signal transducer and activator of transcription 1	Growth signaling	Pro/Anti	−2.63	7.37 × 10^−7^
TAP1	transporter 1, ATP-binding cassette, sub-family B (MDR/TAP)	ABC transporter/multidrug resistance	NK	−1.05	0.04
TAP2	transporter 2, ATP-binding cassette, sub-family B (MDR/TAP)	ABC transporter/multidrug resistance	NK	−2.4	3.83 × 10^−5^
TAPBP	TAP binding protein (tapasin)	MHC I/associated glycoprotein	NK	−1.77	0.0002
TDGF1	teratocarcinoma-derived growth factor 1	Growth EGF signaling	Pro	1.36	0.0076
TRIM22	tripartite motif containing 22	Innate Immunity/Ant-viral	Pro	−0.94	0.018
TRIM31	tripartite motif containing 31	SRC induced growth regulator	NK	1.29	0.0461
TRIM38	tripartite motif containing 38	Innate Immunity	NK	−1.04	0.0218
UBA7	ubiquitin-like modifier activating enzyme 7	Activates ubiquitin ISGylation	NK	−2.03	0.0088
UBE2L6	ubiquitin-conjugating enzyme E2L 6	Ubiquitation of FLT3	Pro	−2.13	0.0004
USP18	ubiquitin specific peptidase 18	Negative regulator of interferon signaling	Pro	−3.73	9.57 × 10^−8^
XAF1	XIAP associated factor 1	Apoptotic protein(s) inhibitor	Anti	−4.07	0.002

DRGs between VECs cultured in CM miR193a and CM mimic CTR implicated in the enriched pathways. Table depicts list of genes involved in the significant enriched pathways from BioPlanet and GO Biological Process on the Enrichr website by uploading DRGs obtained by Transcriptome Analysis Console (TAC). It also depicts gene function and their role in angiogenesis as well as fold changes (FC) and adjusted *p*-values (FDR *p*-value). NK; not known.

## Data Availability

All data supporting the findings of this study are available within the article and its Supplementary file or from the corresponding author upon reasonable request.

## Supplementary Materials

The following are available online at https://www.mdpi.com/article/10.3390/cells11192967/s1, Figure S1: MiRNA193a sequence in FASTA format and miRNA193a-3p sequence, Figure S2: Immuno-histochemical staining of spheroids transfected with mimic control and miR193a-3p, Table S1: Changes in genes using BioPlanet, GO Biological Process, KEGG, GO Cellular Component, GO Molecular function and MSigDB Hallmark.
