# Supplementary material for: Micro-RNA193a-3p Inhibits Breast Cancer Cell Driven Growth of Vascular Endothelial Cells by Altering Secretome and Inhibiting Mitogenesis: Transcriptomic and Functional Evidence
Source: Cells. 2022 Sep 23;11(19):2967. doi: 10.3390/cells11192967 (PMC9562882; doi:10.3390/cells11192967)
Supplement: Supplementary file 1 [file cells-11-02967-s001.zip › Supplementary data_.pdf]

microRNA 193a (MIR193A)	5' – CGAGGATGGGAGCTGAGGGCTGGGTCTTTGC GGGCGAGATGAGGGTGTCGGATC**AACTGGC CTACAAAGTCCCAGT**TCTCGGCCCCCG – 3'
microRNA 193a-3p	5' – **AACTGGCCTACAAAGTCCCAGT** – 3'

Figure S1: MiRNA193a sequence in FASTA format and miRNA193a-3p sequence.

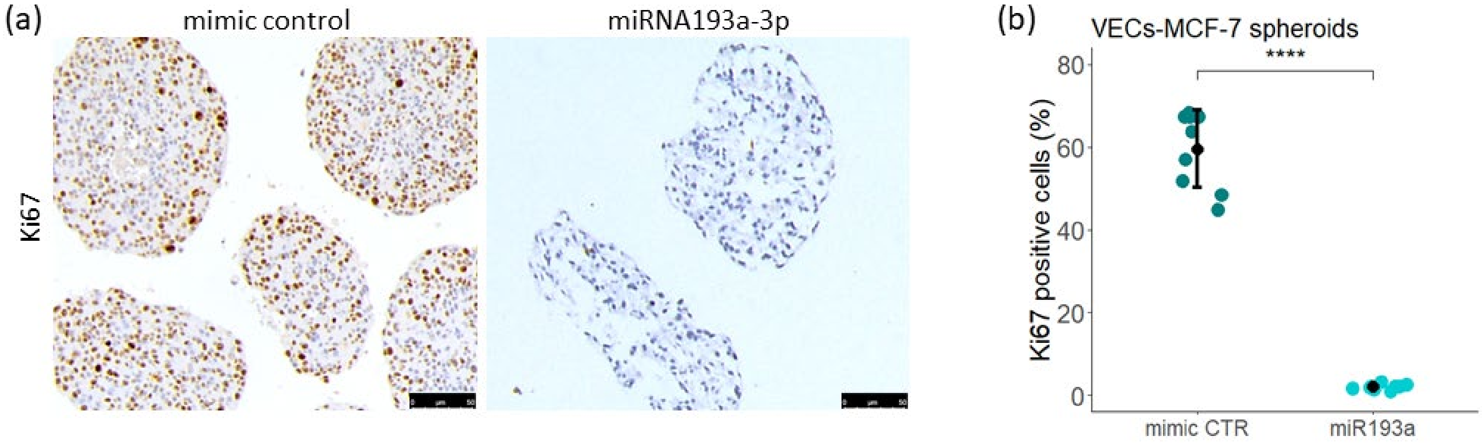

Figure S2: Immuno-histochemical staining of spheroids transfected with mimic control and miR193a-3p. (a) Representative images of MCF-7+VEC spheroids sections stained with Ki67, proliferative staining. (b) Significantly, decreased expression of Ki67 reflects the decreased cell proliferation in spheroids transfected with miRNA193a. $p < 0.005$*** compared to the miRNA control. Ki67 positive cells were 60% ± 9 in mimic CTR spheroids and 2% ± 0.6 in miR193a spheroids. The percentages were calculated by ImageJ software and compared to the total number of cells within the spheroids. Scale bar, 50µm.
